# The Potential Roles of the G1LEA and G3LEA Proteins in Early Embryo Development and in Response to Low Temperature and High Salinity in *Artemia sinica*

**DOI:** 10.1371/journal.pone.0162272

**Published:** 2016-09-07

**Authors:** Wei Zhao, Feng Yao, Mengchen Zhang, Ting Jing, Shuang Zhang, Lin Hou, Xiangyang Zou

**Affiliations:** 1 College of Life Sciences, Liaoning Normal University, Dalian, China; 2 Department of Biology, Dalian Medical University, Dalian, China; Zhejiang University College of Life Sciences, CHINA

## Abstract

Late embryogenesis abundant proteins (LEA) are stress resistance-related proteins that play crucial roles in protecting against desiccation, cold and high salinity in a variety of animals and plants. However, the expression pattern, distribution and functions of LEA proteins in the post-diapause period of *Artemia sinica*, and under high salinity and low temperature stresses, remain unknown. In this study, the complete cDNA sequences of *the group 1 LEA* (*As*-*g1lea*) and *group 3 LEA* (*As*-*g3lea*) genes from *A*. *sinica* were cloned. The expression patterns and location of *As*-G1LEA and *As*-G1LEA were investigated. The protein abundances of *As*-G1LEA, *As*-G3LEA and Trehalase were analyzed during different developmental stages of the embryo and under low temperature and high salinity stresses in *A*. *sinica*. The full-length cDNA of *As*-*g1lea* was 960 bp, encoding a 182 amino acid protein, and *As*-*g3lea* was 2089 bp, encoding a 364 amino acid protein. *As*-*g1lea* and *As-g3lea* showed their highest expressions at 0 h of embryonic development and both showed higher relative expression in embryonic, rather than adult, development stages. The abundances of *As*-G1LEA, *As-*G3LEA and trehalose were upregulated under low temperature and downregulated under high salinity stress. These two genes did not show any tissue or organ specific expression. Our results suggested that these LEA proteins might play a pivotal role in stress tolerance in *A*. *sinica*

## Introduction

*Artemia sinica*, also known as the brine shrimp, is a small, primitive crustacean that lives in inland hyperosmotic salt lakes and coastal salt works in China. The *Artemia* was first reported in 1755 by Schlosser, who published an article about *Artemia’s* morphology. It was named *Artemia salina* by Leach in 1819 [1]. *Artemia* has high unsaturated fatty acid, protein and vitamin contents; therefore, it is used as the initial feed of the larvae of marine fishes, prawns and crabs. *Artemia* can produce diapause embryos, known as cysts, whose development and metabolism are suspended; these cysts are able to remain viable for many years without water [2,3]. Therefore, *Artemia* is a good model organism in which to study different fields of embryo development, genetics, molecular biology, and temperature and high salinity stress responses [4,5].

Lack of water causes many forms of cellular damage and LEA proteins play crucial roles in protecting organisms against desiccation damage [6]. This important class of stress resistance related proteins are involved in desiccation tolerance in many organisms [7]. LEA proteins are categorized into at least seven groups by the similarities of their deduced amino acid sequences. Most LEA proteins are characterized by high hydrophilicity and thermostability [8]. In the 1980s, Dure et al. first reported the existence of an LEA gene in developing seeds of cotton [9]. Subsequently, LEA proteins were found in a number of seeds, pollen and other vegetative tissues of plants. In recent years, scientists have identified LEA proteins in other organisms, such as nematodes, bdelloid rotifers, algae, lichens, archaea, microbes and arthropods, such as *Artemia* [10,7].

Group 1 LEA proteins (G1LEA) are highly hydrophilic and contain the 20 amino acid repeat motif TRKEQ[L/M]G[T/E]EGY[Q/K]EMGRKGG[L/E]. This motif may be present in one to four copies arranged in tandem in plant species, and in up to eight copies in other organisms [11]. It has not been reported in any animal other than *Artemia* [12]. LEA proteins are extremely hydrophilic, which helps to prevent damage by water stress [13]. So far, there is no direct evidence of the function of group 1 LEA proteins. In vitro, group 1 LEA proteins protected citrate synthase against drying, accompanied by a significantly enhanced trehalose content[14,12]. During seed development, LEA proteins slow down water loss, acting as a buffer [15,16]. In animals, it is likely be beneficial for intracellular glass formation [6].

Group 3 LEA proteins (G3LEA) are characterized by a repeat motif of 11 amino acids. This group of proteins has varied molecular masses as a consequence of the number of repetitions of this 11-mer motif [17,11]. The 11-mer in group 3 LEA proteins always exists as an amphipathic *-*helix in the prediction of the secondary structure; however, the proteins are largely in a random coil conformation in solution [18]. Experimental results showed that the structure of this protein may depend on the drying rate and that this kind of structural change is reversible [19,20].

Trehalose a kind of non-reducing disaccharide which can protects membranes and proteins from desiccation damage by replacing structural water, and helps to form an intracellular organic glass to stabilize cellular structures[21,22]. Trehalase can degrade trehalose to the glucose involved in energy metabolism. A group 3 LEA protein was found to provide structural protection for the mitochondrial membrane because of its alpha helical structure in pea (*Pisum sativum*) [20,23]. The cryopreservation of liposomes by LEA proteins was decided to the lipid composition of the bilayer and external trehalose can significantly decreased damage to all liposomes tested [24]. Group 3 LEA proteins also decrease the interaction aggregation of denaturing proteins and then generate space filling filaments that enable cells to confront water content decreases, acting as "molecular shields” [25,26]. It is unclear how LEA proteins play these such diverse roles, including guarding against salt and cold stress, chilling and freezing, and water stress; however, their diverse functions are undoubtedly associated with their hydrophilicity and limited secondary structure [12,27,28].

Many studies of LEA proteins have focused on plants; however, the expression pattern, distribution and the role of LEA proteins in post-diapause of *A*. *sinica* and in the response to high salinity and low temperature stress, remain unknown. In the present study, the full-length cDNA sequences representing the *A*. *sinica g1lea* and *g3lea* genes were cloned by rapid amplification of cDNA ends (RACE). The expression patterns and expression location of *g1lea* and *g3lea* genes in different embryonic development stages of *A*. *sinica* were investigated by quantitative real-time PCR (qPCR) and immunofluorescence labeling. The expression level of the *As-*G1LEA and *As-*G3LEA proteins during different developmental stages and in response to high salinity and low temperature stress were analyzed by western blotting. Our aims were to further understand the role of the *g1lea* and *g3lea* genes in diapause embryo restarting and in response to high salinity and low temperature stress in the early embryonic development of *A*. *sinica*.

## Materials and Methods

### Animal preparation

No specific permits were required for the collection of samples and field studies using *Artemia* cysts. The location was not privately owned or protected in any way, and the field studies also did not involve endangered or protected species. We confirm that the salt lake and land on which we conducted our study on was not privately owned or government protected.

*Artemia sinica* cysts were collected from the salt lake of Yuncheng in Shanxi Province, China. The cysts were stored at −20C and incubated at 30C in filtered seawater with 28‰ salinity and an illumination intensity of 1000 lx. *Artemia sinica* cyst samples (~50 mg) were collected at different developmental stages (5, 10, 15, 20 and 40 h, and 3, 5, and 7 d) for subsequent experiments. For the low temperature tolerance and salinity stress conditions, *A*.*sinica* cysts (20h old) were maintained at 30C, 28‰ salinity natural seawater for 44 h and as the control group. While *A*. *sinica* at the same stage (20 h) were held at 25, 20, 15, 10 or 5C. For the salinity stress condition, *A*. *sinica* cysts at the same stage (20 h) were treated with high salinity (50‰, 100‰, 150‰ and 200‰).

### Cloning of *As-g1lea* and *As-g3lea*

Total RNA from *A*. *sinica* cysts (0 h) was extracted according to the kit instructions of RNAiso Plus (Takara, Dalian, China) and reverse transcribed into cDNA using an oligo (dT) primer and MLV reverse transcriptase (Takara). Specific primer sequences were designed based on the partial sequence of *Artemia franciscana G1LEA and G3LEA* and synthesized by Shenggong (Shanghai, China) using Premier 5.0 (Table 1). The PCR reaction conditions were as follows: initial incubation at 94C for 5 min; followed by 30 cycles of denaturation at 94C for 30 s, annealing at 55C for 30 s, elongation at 72C for 1 min; and a final incubation at 72C for 10 min. The full-length cDNAs of *As-g1lea* and *As-g3lea* were obtained using 3′-RACE (Takara) and 5′-RACE kits (Clontech, Chicago, USA), according to the manufacturer's instructions. The resultant PCR products were recovered and cloned into the PEASY-T1 vector (Quanshijin, Beijing, China) and sequenced. The 3′ and 5′ fragments were spliced together to obtain the full-length cDNA sequence using DNAman 6.0 (Lynnon Biosoft). The nucleotide sequence was submitted to GenBank (*As-g1lea* GenBank accession number: AMQ80946.1, *As-g3lea* GenBank accession number: KU851934).

**Table 1:** Oligonucleotide primers used in this study.

Primer	Sequence (5-3)	Direction
*As*-*g1lea*F	GGTGACATTTGCTGCTTACT	Forward
*As*-*g1lea*R	ATTTCTATCGTTTATTTGGA	Reverse
3'Ou-*As-g1lea*	CATGCTAAGCCACCAAGAAG	Forward
3'In-*As*-*g1lea*	AACAGTTGGGTCACGAAGGT	Forward
5'*As*-*g1lea*	CAGGCAGCCATTGGGCAGTAAGG	Reverse
*As*-*g3lea*F	ATGCCAAAAGCAGCAGCTAAAG	Forward
*As*-*g3lea*R	CTATTCAGGGTTTTCTT	Reverse
3'Ou-*As*-*g3lea*	ATGAAAGGTTAATCCACAAGAG	Forward
3'In-*As*-*g3lea*	GTTCACGATCTCAAGTTGCTGG	Forward
5'*As*-*g3lea*	TTTGTAGAGTCAATAGTCTTGTCAT	Reverse
RT-*g1lea*F	AGAAACCAAACGGGTAAACCGAATC	Forward
RT-*g1lea*R	CACCTCTTTGTCCAGCTTCTTGG	Reverse
RT-*g3lea*F	TCCGGTAAGCACGCGTATGATTCGAC	Forward
RT-*g3lea*R	CGGACTTCACCAGATGCTGTGCTAA	Reverse
*gapdh*-F	GGTCGTGACTTGACGGACTATCT	Forward
*gapdh*-R	AGCGGTTGCCATTTCTTGTT	Reverse
ORF-*g1lea*F	ATGAGTGAACAGGGAAAGCTAAG	Forward
ORF-*g1lea*R	TTATTGTTGTCTAGCGAGACCTC	Reverse
ORF-*g3lea*F	ATGCCAAAAGCAGCAGCTAAAG	Forward
ORF-*g3lea*R	CTATTCAGGGTTTTCTTTTGG	Reverse

### Bioinformatic analysis

The cloned nucleotide sequences were analyzed for identity and similarity using the NCBI (National Center for Biotechnology Information) online Search Tool (BLASTX) (http://www.ncbi.nlm.nih.gov/), and the open reading frames (ORFs) were identified using the online ORF Finder program at the NCBI (http://www.ncbi.nlm.nih.gov/projects/gorf/). The functional domains and structure of the proteins were predicted using prosite tools of ExPASy (http://prosite.expasy.org/prosite.html/) and SMART (http://smart.embl-heidelberg.de/). The molecular weight and theoretical isoelectric point of the proteins were estimated using ProtParam (http://web.expasy.org/protparam/), and the single transmembrane region was analyzed using TMHMM (http://www.cbs.dtu.dk/services/TMHMM/). The LEA proteins were analyzed for the presence of a signal peptide using SignalP 4.1 (http://www.cbs.dtu.dk/services/SignalP/). Protscale (http://web.expasy.org/protscale/) was used to detect hydrophobicity and hydrophilicity.

### Expression pattern detection by qPCR

#### Expression levels during different developmental stages

The mRNA of the two genes in different developmental periods from *A*. *sinica* was extracted and reversed transcribed into cDNA (see section “Cloning of As-g1lea and As-g3lea”). Three pairs of specific primers were designed according to the full-length sequences of *Asg1lea*, *As-g3lea* and *gapdh* gene, which was used as a normalization control for each RNA sample (Table 1). The qPCR was performed in triplicate for each sample using the SYBR Premix Ex Taq (Takara) and the Takara TP800 detection system. The qPCR reaction conditions were as follows: 95C for 30 s; followed by 40 cycles each of 95C for 5 s, 57C for 30 s, 95C for 15 s, 60C for 30 s and 95C for 15 s. Dissociation analysis was performed at the end of each reaction to confirm that only one PCR product was amplified. Gene expression data were analyzed using Thermal Cycler Dice Real Time System Software (Takara) and quantified using the comparative cycle threshold (Ct) method (2^−ΔΔCt^ method) based on Ct values for both *As-g1lea* and *As-g3lea* and *gapdh* to calculate the relative fold increase [29]. Data obtained from qPCR analysis were analyzed by least square difference (LSD), and significance was set at *P*< 0.05, as assessed by a t-test using SPSS 16.0 software.

#### Salinity and temperature stress assays

Total RNAs were collected from brine shrimp treated with different salinities and temperatures and qPCR was performed on the samples using the primers and reaction conditions have been described detailed in the “Expression levels during different developmental stages” section

### Purification and expression of the As-G1LEA and As-G3LEA protein

The upstream and downstream primers to amplify the full-length *As-g1lea* gene, with enzyme sites BamHI and SalI, were designed using Primer5 software (Table 1). The product and pET-30a fragments were purified after double enzyme digestion at 37C for 4 h. Both the recombinant PCR products and pET30a digested with the enzymes BamHI and SalI were ligated together using T4 DNA ligase (Takara) at 16C overnight. The resulting expression vector, pET-30a-G1LEA, was sequenced by Shenggong (Shanghai, China).

The recombinant expression plasmid pET-30a-G1LEA was transformed into *Escherichia coli* BL21(DE3), and the conditions for expression and induction of the protein were as follows: 1 mM IPTG for 3 h at 37C, 1 mM IPTG for 3 h at 30C, 0.25 mM IPTG for 3 h at 37C, and 0.25 mM IPTG for 3 h at 30C. Cells were collected by centrifugation at 7500 rpm for 5 min and then washed twice with PBS. One volume of SDS–PAGE loading buffer was added to each sample, which was then boiled for 8 min. The best of the four induction conditions for large-scale purification by ultrasonication was based on SDS–PAGE analysis of the supernatant and the cell pellet. The recombinant *As*-G1LEA protein was expressed in a 1 L culture of *E*. *coli* BL21 (DE3), induced with 1 mM IPTG at 37C for 3 h. Cells were collected by centrifugation at 10000 rpm for 5 min at 4C and the sediment was resuspended in equilibration buffer containing 20 mM Tris-HCl (pH 8.5), 150 mM NaCl and 20 mM imidazole, before being lysed by ultrasonication. Purification of the *As*-G1LEA recombinant protein was accomplished using a HisTrap FF crude (GE Healthcare), following the supplier’s protocol. The only differences in the washing buffer, elution buffer, and equilibration buffer were the imidazole concentrations: 10 mM, 20 mM, 40 mM, 60 mM, 80 mM and 100 mM, respectively. The protein was dialyzed into 40 mM Tris-HCl and then freeze dried by lyophilizer (Japan). *As-g3lea* gene purification and expression was same as that for *As-g1lea*, and was best purified at 40mM Tris-HCl.

### Production of polyclonal antibodies

All studies involving rabbits were approved by the Animal Care and Use Committee of Dalian Medical University, Dalian, Liaoning, China. Eight-week-old female New Zealand rabbits (Specific pathogen Free, SPF) were obtained from the Experimental Animal Center of Dalian Medical University. All experimental procedures were conducted in accordance with institutional guidelines for the care and use of laboratory animals. Polyclonal antibodies directed against the *As-*G1LEA and *As-*G3LEA recombinant proteins were prepared in the SPF rabbits. We extracted 1 ml blood from the ear marginal vein of the rabbit as a negative control before the first injection. Rabbits were immunized every ten days by multipoint intradermal injections. For the first immunization, the purified protein (600 g/mL) was emulsified with an equal volume of Freund's complete adjuvant. For the four subsequent immunizations, 300 g/mL purified protein was emulsified with an equal volume of Freund's incomplete adjuvant. The antiserum was collected by centrifugation at 12000 rpm for 20 min. An enzyme linked immunosorbent assay (ELISA) was used to measure the concentration and western blotting was used to determine the specificity of the antibody for the purified protein.

### Preparation of paraffin sections and immunofluorescence labeling

Brine shrimp were collected at different embryonic development stages (0h, 15h, 3d) and prepared as paraffin sections at a thickness of 8μm.The paraffin sections were dewaxed and hydrated before being fixed in 4% paraformaldehyde for 10 min, treated with 0.2% TritonX-100 with PBS for 15 min, and blocked with 4% BSA for 2 h. The rabbit anti-LEA1 polyclonal antibody was diluted 1:50 with 4% BSA, followed by overnight incubation at 4C. The sections were washed the next day and incubated with Cy3 AffiniPure goat anti-rabbit IgG (H + L) antibody for 2 h at 37C in dark (The rabbitanti-G3LEA polyclonal antibody was diluted 1:50 and incubated with FITCAffiniPure goat anti-rabbit IgG (H + L) antibody). The 4′,6-Diamidino-2-phenylindole dihydrochloride (DAPI) cell nuclear blue fluorescent probe was added onto the samples and incubated for 10 min. Finally, the sections were sealed with antifade mounting medium and examined under a confocal microscope (ZEISS LSM 710).

### Western blotting

Total proteins were extracted from brine shrimp at different developmental stages (0, 5, 10, 15, 20, 40 h and 3 d), different temperatures and salinity stresses using RIPA lysis buffer and quantified by the BCA method. The proteins were subjected to SDS-PAGE, transferred onto polyvinylidene difluoride membranes, and then blocked with 5% non-fat powdered milk for 1 h at room temperature. Rabbit anti-Trehalase (XinYu,shanghai) 1:500 with PBST. Rabbit anti-*As*-G1LEA and anti-*As*-G3LEA polyclonal antibodies are diluted 1:200 and 1:100 with PBST, respectively, and an anti-GAPDH antibody was diluted 1:500 with PBST as a control group, and incubated with the membranes overnight at 4C. The following day, the membranes were washed three times with PBST for 10 min each, incubated with horseradish peroxidase (HRP)-conjugated goat anti-rabbit IgG antibody for 1 h at 37C, and then washed with PBST three times and PBS once. The reactive protein bands on the membranes were visualized using the ECL reagent (Beyotime, Shanghai, China). The membranes were exposed to an X-ray film in a darkroom. The western blotting membranes were photographed, and the images were analyzed using Image J software. Image grayscale analysis in the Image J software was used to convert the data to column charts. Intensities of *As*-G1LEA-specific bands and *As*-G3LEA-specific bands were normalized against the GAPDH-specific bands.

## Results

### Cloning and bioinformatic analysis of *As-G1LEA and As-G3LEA*

**Fig 1:**
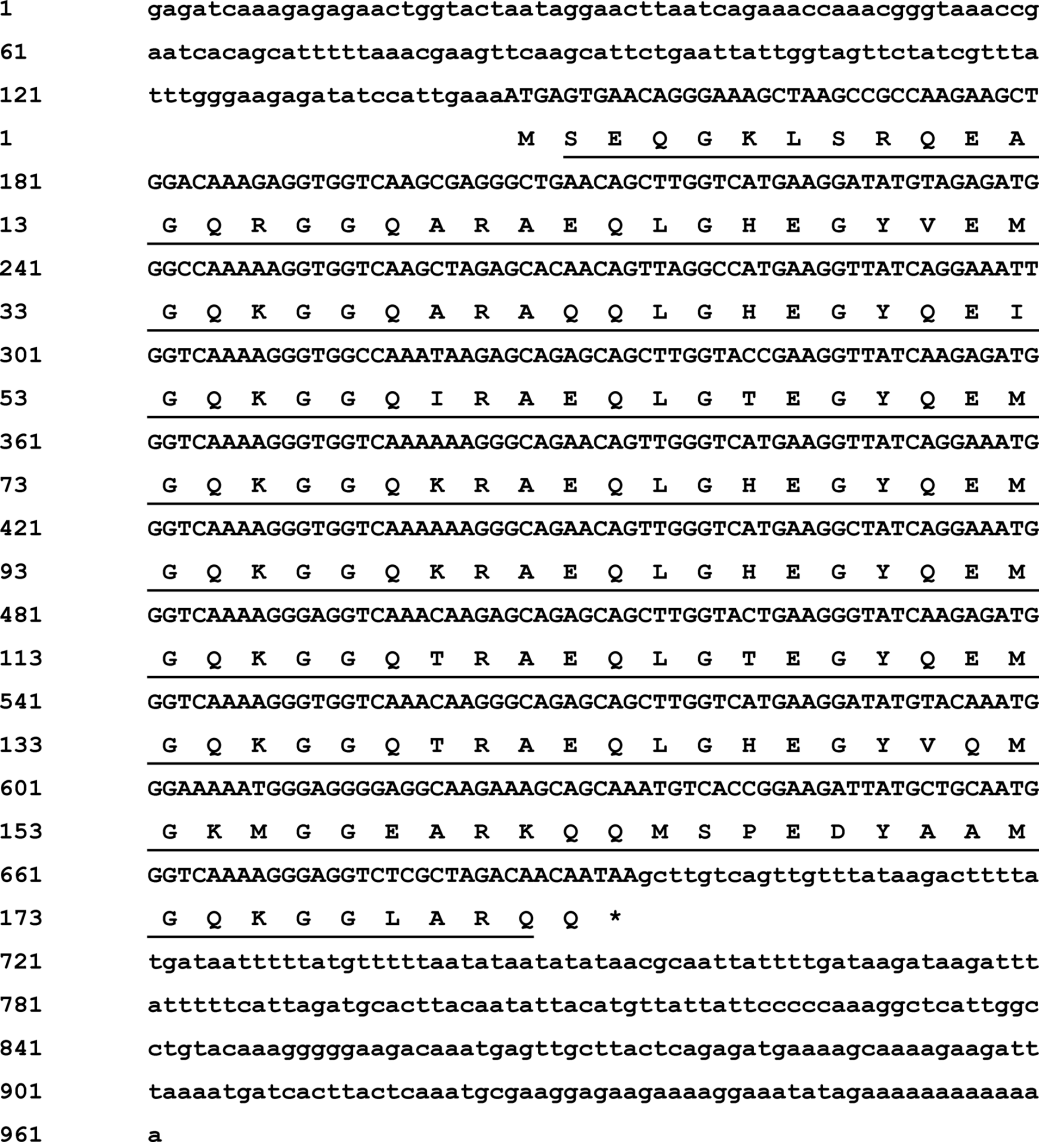
Sequence and structure of *As-g1lea*. Sequence analysis of the cDNA and predicted peptide sequences of *As-g1lea*. Nucleotide and amino acid sequence numbers are shown to the left and the right, respectively. Sequences underlined in black straight lines are the LEA_5 domain.

The full-length cDNA of *As-g1lea* from *A*. *sinica* (GenBank accession number: AMQ80946.1) was obtained by using 3′ and 5′ RACE technology. It was 960 bp, with a 549 bp ORF, a 144-bp 5′-UTR and a 267-bp 3′-UTR (Fig 1). SignalP 3.0 analysis showed that *As-g1lea* had no signal peptide. The hydrophobicity analysis of *As-g1lea* indicated that the protein is hydrophilic.

**Fig 2:**
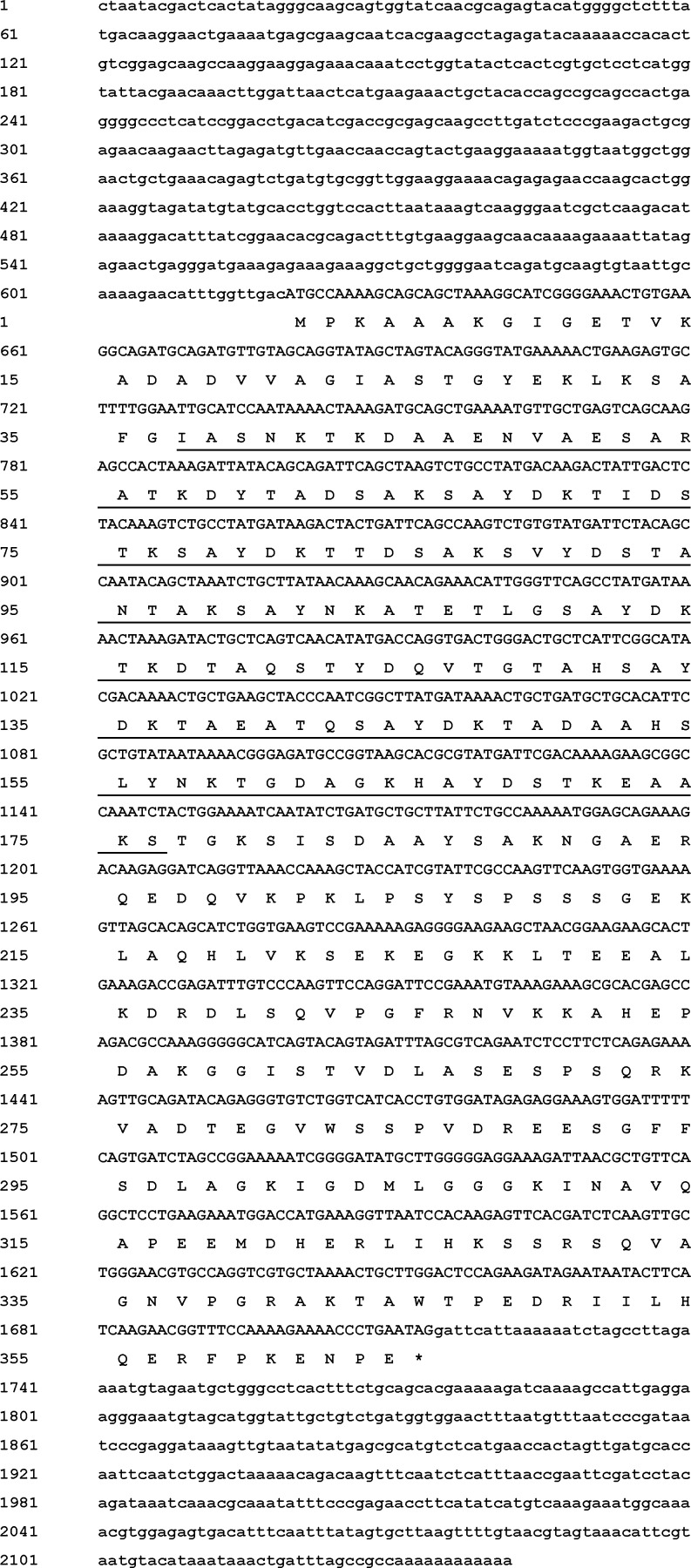
Sequence and structure of *As-g3lea*. Sequence analysis of the cDNA and predicted peptide sequences of *As-g3lea*. Nucleotide and amino acid sequence numbers are shown to the left and the right, respectively. Sequences underlined in black straight lines are the LEA_4 domain.

The full-length cDNA of *As-g3lea* was obtained by using 3′ and 5′ RACE technology. It was found to be 2089 bp, with a 1095 bp ORF, a 566-bp 5′-UTR and a 428-bp 3′-UTR (Fig 2). Signal P 3.0 analysis showed that *As-g3lea* had no signal peptide. The hydrophobicity analysis of *As-g3lea* indicated that the protein is hydrophilic. The full-length nucleotide sequence was submitted to GenBank with the accession number KU851934.

### Analysis of As-g1lea and As-g3lea expression by qPCR

**Fig 3:**
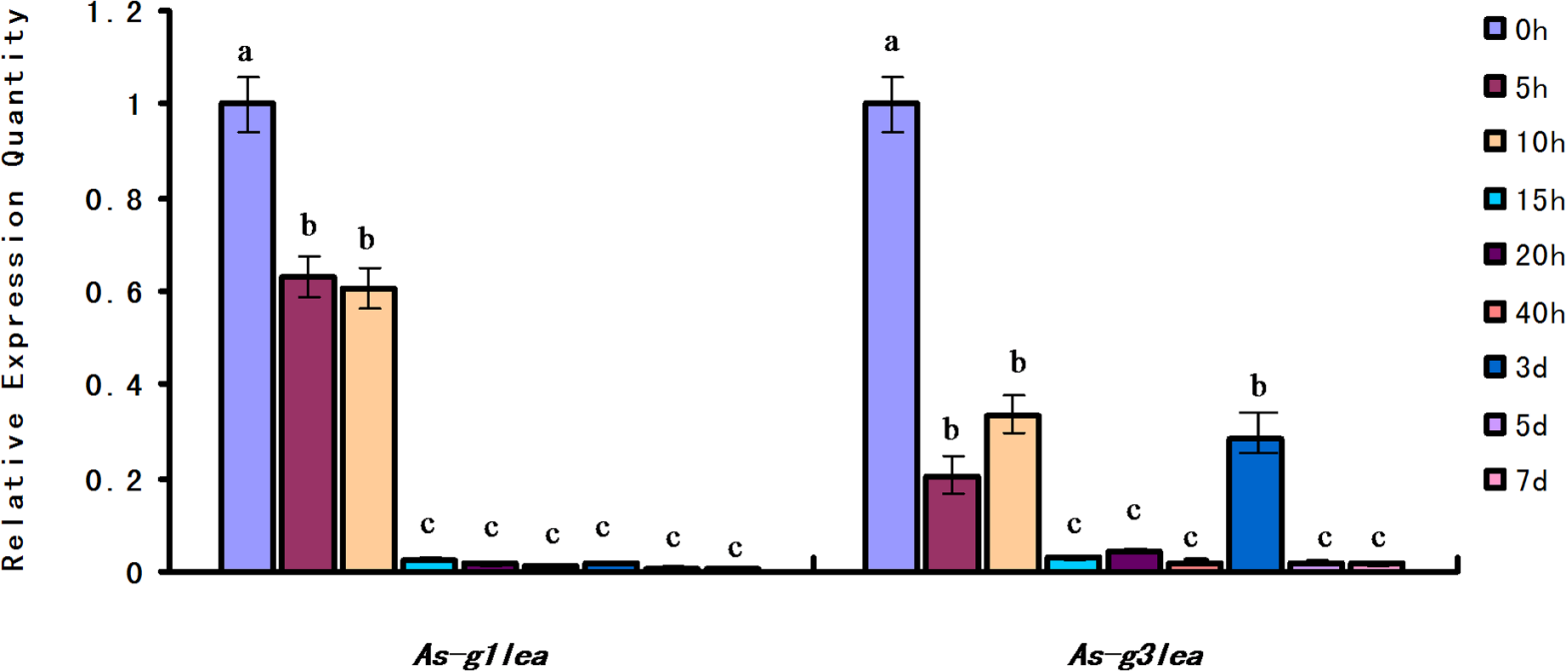
Expression of *As-g1lea* and *As-g3lea* mRNA at different developmental stages. The mRNA expression levels of *As-g1lea*, *As-g3lea* and *gapdh* were measured at certain common developmental stages using quantitative real-time PCR, and data are presented as means ± SD of triplicate experiments. The developmental stage of 0 h was set as the control group. Significant differences at different development stages (*P*< 0.05) were analyzed by one-way analysis of variance (ANOVA) and are indicated by lowercase letters (a, b, c and d).

QPCR analysis of *As-g1lea* expression levels in the early embryonic development of *A*. *sinica* (0 h to 10 h) showed significantly higher expression compared with adult *Artemia*. The expression trend of *As-g1lea* showed a gradual decrease throughout development. The expression of *As-g3lea* at 0 h was higher than the others, and at 5h stage, it declined, but increased again at 10h and 3d (Fig 3).

**Fig 4:**
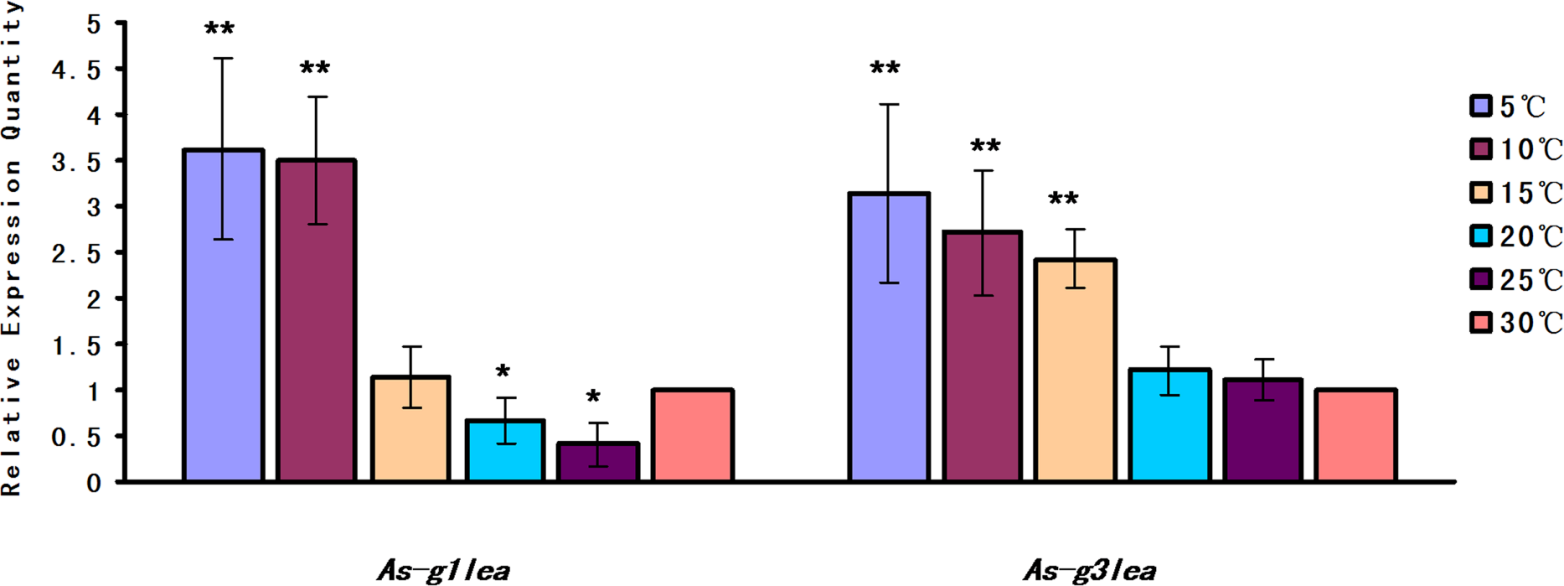
Relative expression of *As-g1lea* and *As-g3lea* mRNA in response to low temperature challenge. The control group was treated at 30C, and the mRNA expression levels of *As-g1lea*, *As-g3lea* and *gapdh* were measured using quantitative real-time PCR 24 h after being incubated at the indicated temperature. Data are presented as the mean ± SD of triplicate experiments. Highly significant differences between the experimental and control groups are indicated with **P < 0.01, while significant difference are indicated with * 0.01 < *P* < 0.05.

*As-g1lea* and *As-g3lea* transcript levels increased significantly upon treatment by lower temperatures (25, 20, 15, 10 and 5C) compared with the control group maintained at 30C. With decreasing temperature, *As*-G1LEA protein expression declined slightly at first and then increased significantly at 5C and 10C, while *As-*G3LEA protein expression gradually increased to its highest level at 5C (Fig 4).

**Fig 5:**
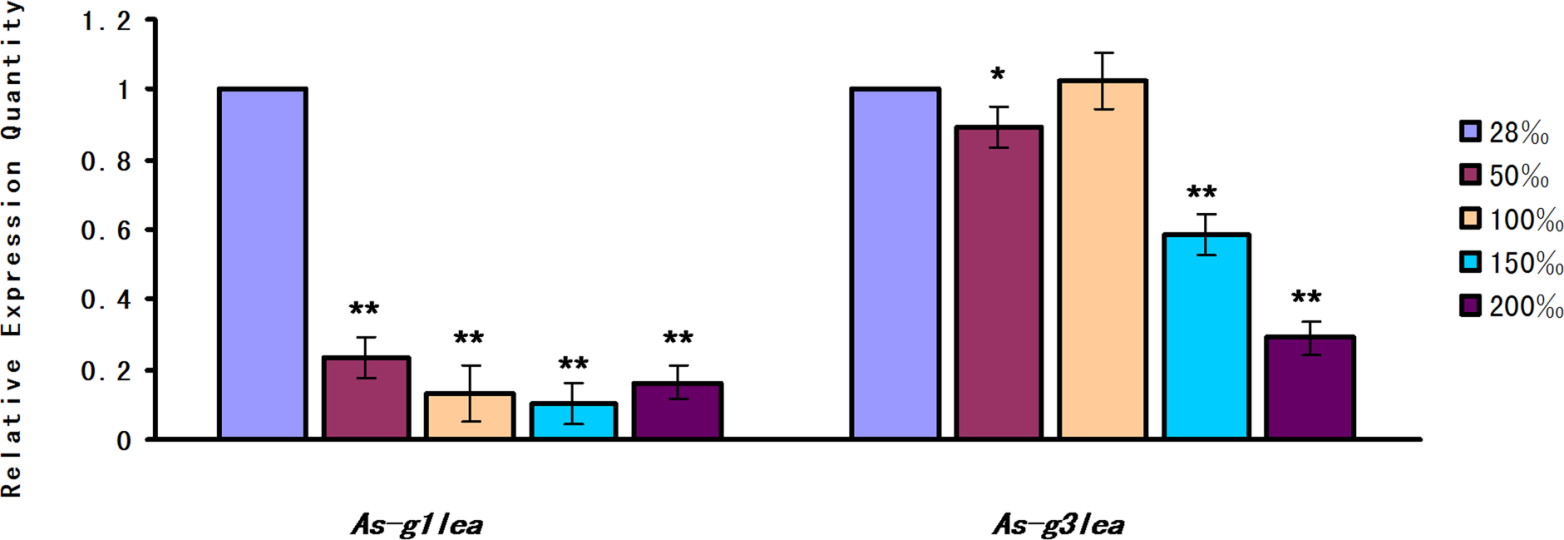
Relative expression of *As-g1lea* and *As-g3lea* mRNA in response to salinity stress. The 28‰ salinity treatment condition served as a control group, and the mRNA expression levels of *As-g1lea*, *As-g3lea* and *gapdh* were measured using quantitative real-time PCR 24 h after challenge with four different salinity concentrations. Data are presented as the means ± SD of triplicate experiments. Highly significant differences between the experimental and control groups are indicated with **P < 0.01, while significant difference are indicated with * 0.01 < *P* <0.05.

The expression level of *As-g1lea* under salinity stress showed a significant decrease between the experimental group and control (28‰) groups. The expression level of *As*-*g3lea* increased gradually from the 28‰ to 100‰, then significantly decreased, reaching its lowest level in the 200‰ salinity group (Fig 5).

### Prokaryotic Expression and Purification of As-G1LEA and *As*-G3LEA Proteins and antibody preparation

**Fig 6:**
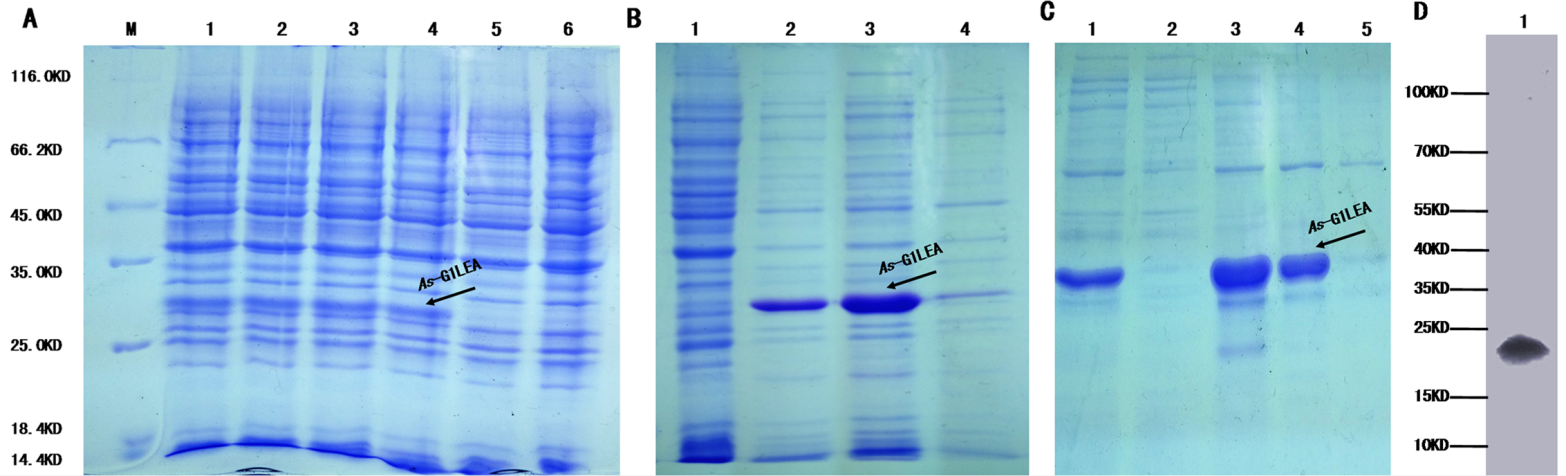
Prokaryotic expression of the *As*-G1LEA protein. (A) Expression analysis of the recombinant *As-*G1LEA protein. Lane M: Protein markers from 10 to 120 kDa. Lanes 1–4 show the expression of the recombinant *As-*G1LEA protein from four induction treatments (1 mM IPTG at 37C, 1 mM IPTG at 30C, 0.25 mM IPTG at 37C, and 0.25 mM IPTG at 30C, respectively). Lane 5: Total proteins from non-induced cells. Lane 6: total proteins from induced cells harboring pET-30a (control). (B) Determination of the solubility of the *As-*G1LEA recombinant protein. Lane 1: Total proteins from non-induced cells. Lane 2: Total *As-*G1LEA recombinant protein. Lane 4: Soluble fraction of the lysate from induced cells harboring pET-30a-G1LEA. Lane 4: Insoluble fraction of the lysate from induced cells harboring pET-30a-G1LEA. (C) Lane 1: Unpurified, induced *As-*G1LEA recombinant protein. Lane 2: Flow-through proteins. Lane 3: 20mM imidazole eluate. Lane 4: 40mM imidazole eluate. Lane 5: 60 mM imidazole eluate. (D) Western blot showing specific binding of the antibody to the *A*. *sinica* protein.

The *As-*G1LEA protein was expressed prokaryotically and purified. Its molecular weight determined as 19 kDa. SDS-PAGE analysis revealed that the recombinant protein was expressed under all four induction conditions (Fig 6A). The first treatment (1 mM IPTG at 37C) was chosen for further research. SDS-PAGE analysis showed that the recombinant protein was present in the soluble fraction isolated from *E*. *coli* BL21 (DE3) (Fig 6B). Using histidine affinity chromatography, the recombinant *As-*G1LEA proteins were purified, and a relatively pure protein sample was obtained elution in 40 mM imidazole (Fig 6C). The molecular mass of the recombinant protein was larger than 19 kDa because of the His-tag. After adding adjuvants to the purified protein and immunizing rabbits, we acquired a polyclonal antibody of rabbit with a valence of 1:256000, as determined by ELISA.

**Fig 7:**
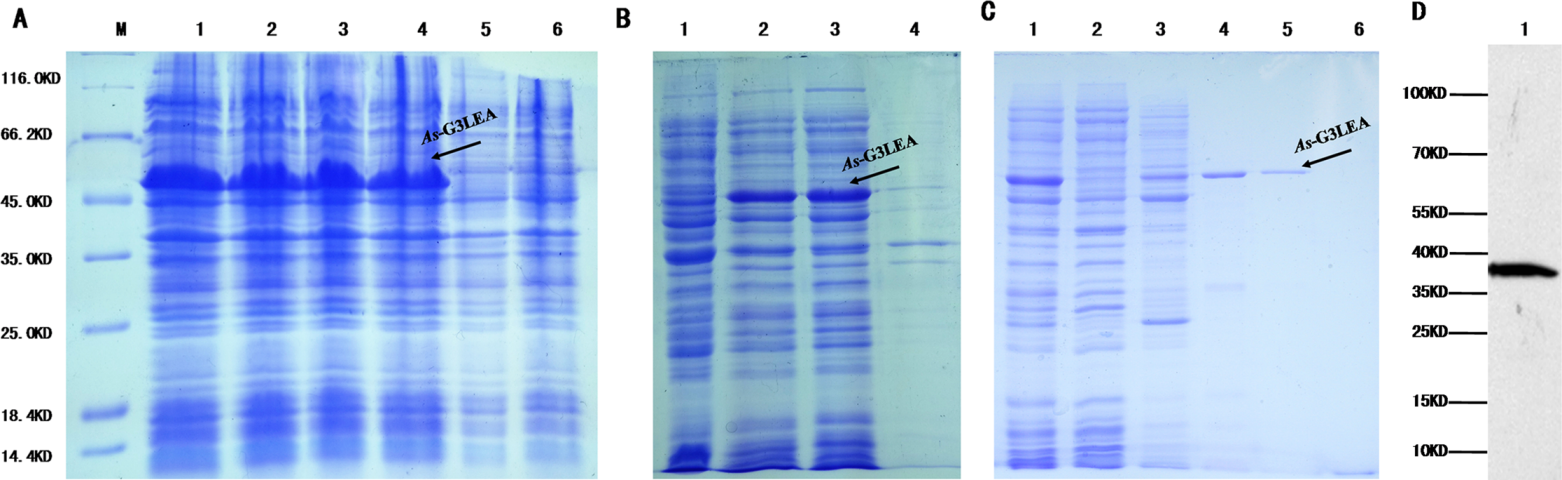
Prokaryotic expression of the *As*-G3LEA protein. (A) Expression analysis of the recombinant *As-*G3LEA protein. Lane M: Protein markers from 10 to 120 kDa. Lanes 1–4 show the expression of the recombinant *As-*G3LEA protein from four induction treatments (1 mM IPTG at 37C, 1 mM IPTG at 30C, 0.25 mM IPTG at 37C, and 0.25 mM IPTG at 30C, respectively). Lane 5: Total proteins from non-induced cells. Lane 6: total proteins from induced cells harboring pET-30a (control). (B) Determination of the solubility of the *As-*G3LEA recombinant protein. Lane 1: Total proteins from non-induced cells. Lane 2: Total *As-G3LEA* recombinant protein. Lane 4: Soluble fraction of the lysate from induced cells harboring pET-30a-G3LEA. Lane 4: Insoluble fraction of the lysate from induced cells harboring pET-30a-G3LEA. (C) Lane 1: Unpurified, induced *As-*G3LEA recombinant protein. Lane 2: Flow-through proteins. Lane 3: 10mM imidazole eluate. Lane 4: 20mM imidazole eluate. Lane 5: 40mM imidazole eluate. Lane 6: 60mM imidazole eluate. (D) Western blot showing specific binding of the antibody to the *A*. *sinica* protein.

The *As-*G3LEA protein was expressed prokaryotically and purified,.Its molecular weight determined as 38 kDa. Among the four treatments, 0.25 mM IPTG at 30C was chosen for further research (Fig 7A). *As-*LEA group 3 proteins exists in a soluble form (Fig 7B). *As-*G3LEA proteins were purified, and a relatively pure protein sample was obtained elution using 40 mM imidazole (Fig 7C). Finally, we acquired a rabbit polyclonal antibody with a valence of 1:128000, as judged by ELISA.

### Analysis of As-G1LEA and As-G3LEA protein expressions by western blotting

**Fig 8:**
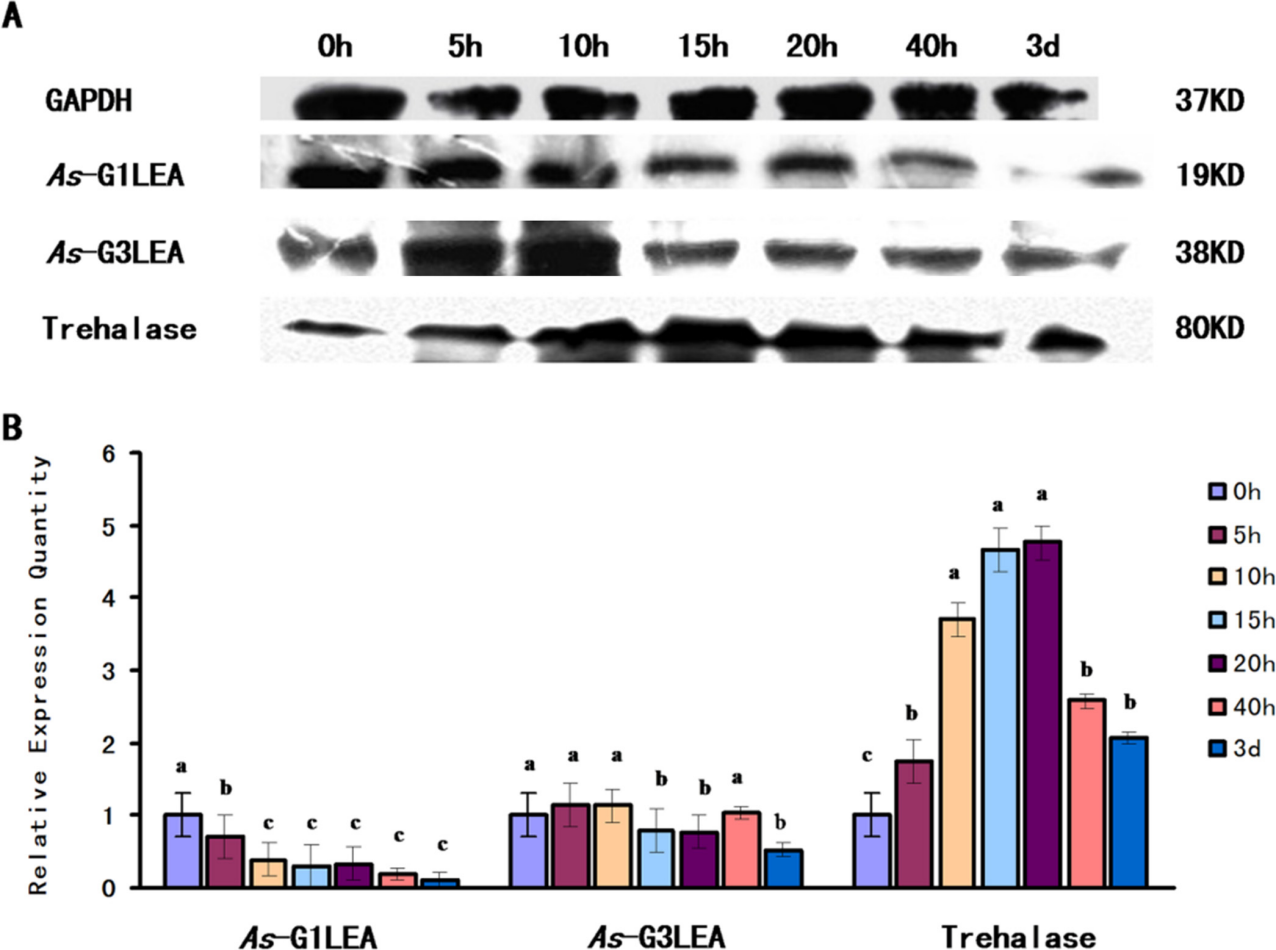
Western blotting analysis of *As*-G1LEA, *As*-G3LEA and Trehalase at different developmental stages. (A) Western blotting analysis of the expression of *As*-G1LEA, *As*-G3LEA and Trehalase proteins at different developmental stages in *A*. *sinic*a. The intensities of the *As*-G1LEA, *As*-G3LEA and Trehalase protein bands were normalized against those of GAPDH. (B–D) Values are expressed as arbitrary units of relative value. The expressions of *As*-G1LEA, *As*-G3LEA and Trehalase proteins at 0 h were used as the control. Significant differences for the different development stages (*P*< 0.05) were analyzed by ANOVA and are indicated by lowercase letters (a, b and c).

The abundance of the *As*-G1LEA protein at different developmental stages in *A*. *sinica* was the highest at the 0 h stage and showed a downward trend during the early developmental stage, reaching its lowest level at 3d. The *As*-G3LEA protein showed the same trend. Trehalase expression levels increased with time and reached a maximum at 20 h, and then began to decrease (Fig 8).

**Fig 9:**
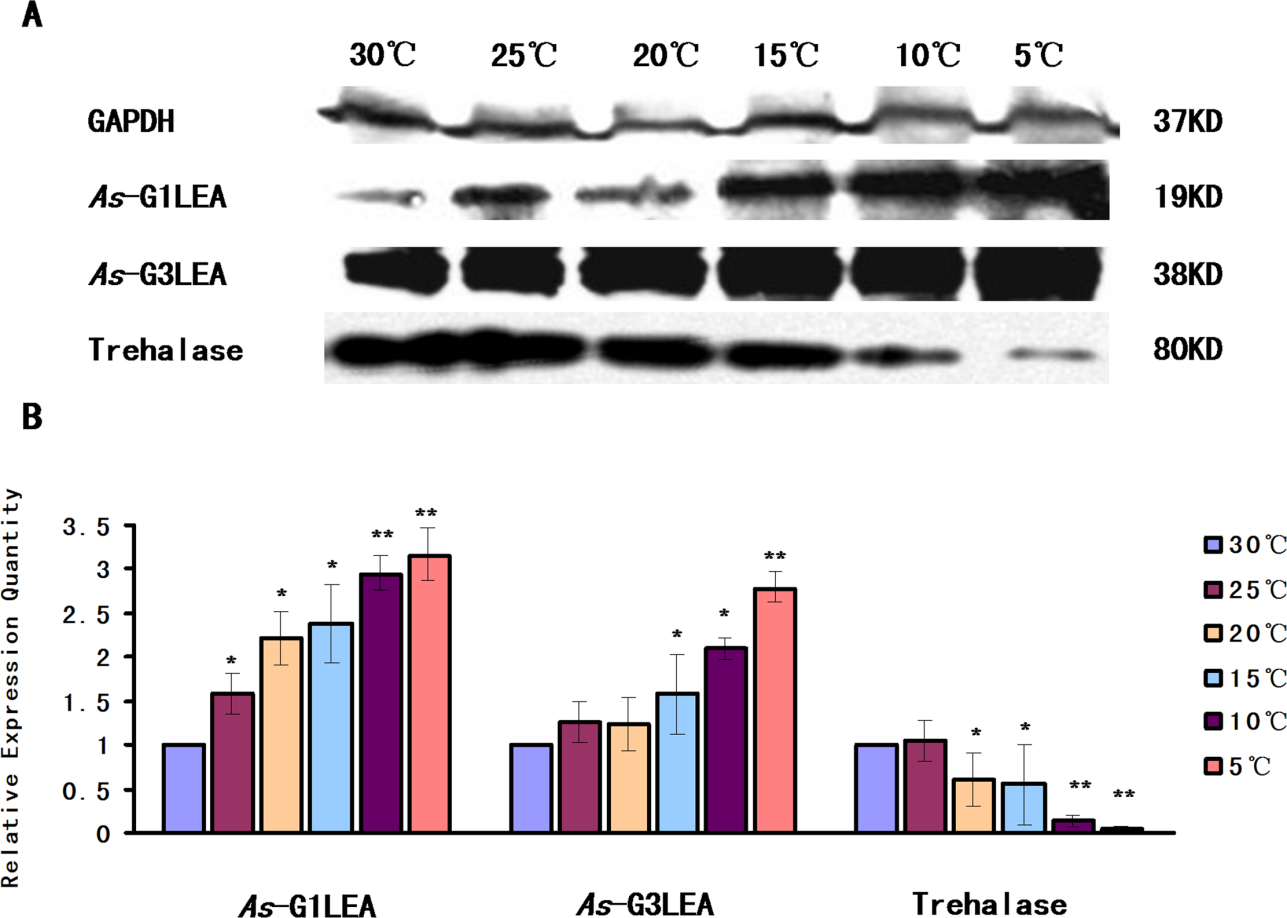
Western blot analysis of *As*-G1LEA, *As*-G3LEA and Trehalase under different temperature challenge. (A) Western blotting analysis of the expression of *As*-G1LEA, *As*-G3LEA and Trehalase proteins at different temperatures in *A*. *sinica*. The intensities of the *As-G1LEA*, *As-G3LEA* and Trehalase protein bands were normalized against those of GAPDH. (B–D) Values are expressed as arbitrary units of relative value. The expressions of *As*-G1LEA, *As*-G3LEA and Trehalase protein at 30C were used as the control. Statistically significant differences are indicated with ** *P* < 0.01, while * represents 0.01 < *P* < 0.05.

In the temperature tolerance assay, the expression level of the *As*-G1LEA protein and *As*-G3LEA protein rose sharply as the temperature decreased from 25C to 5C, achieving their highest levels at 5C. The expression level of Trehalase, which has the opposite trend with the LEA proteins, gradually decreased as the temperature decreased, reach a minimum at 5C (Fig 9).

**Fig 10:**
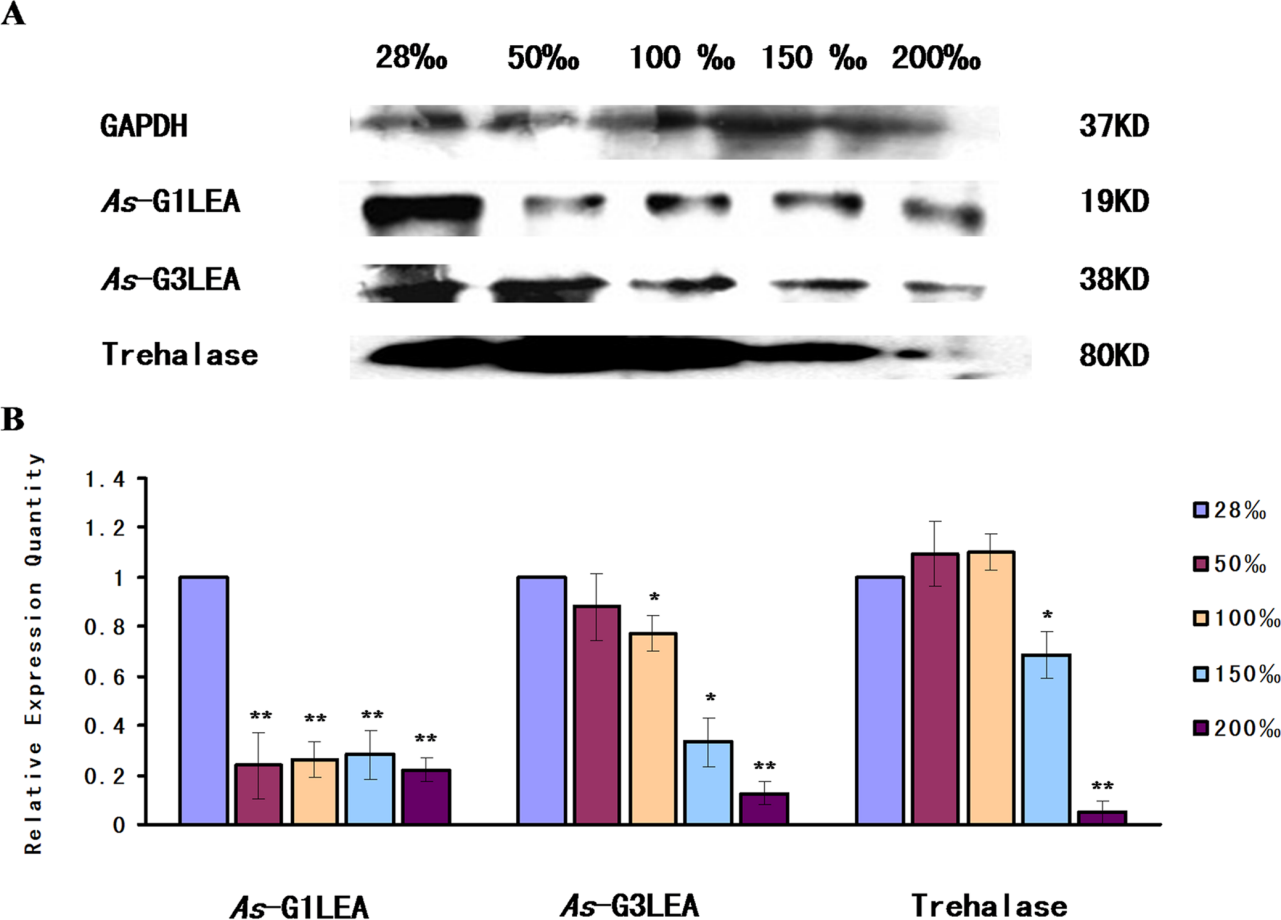
Western blot analysis of *As*-G1LEA, *As*-G3LEA and Trehalase under different salinity stresses. (A) Western blotting analysis of the expressions of *As-G1LEA*, *As-G3LEA* and Trehalase proteins in response to salinity stress in *A*. *sinica*. The intensities of the *As*-G1LEA, *As*-G3LEA and Trehalase protein bands were normalized against those of GAPDH. (B–D) Values are expressed as arbitrary units of relative value. The expressions of *As-*G1LEA, *As-*G3LEA and Trehalase protein at 28‰ salinity were used as the control. Statistically significant differences are indicated with ** *P* < 0.01, while * represents 0.01 < *P* < 0.05.

In the salinity stress assay, under high salinity stress, *As*-G1LEA was present in a lower abundance compared with that in natural seawater (28‰) in *A*. *Sinica*. The abundance of *As*-G3LEA appeared to be downregulated gradually from 50‰ to 100‰ and showed a sudden drop from 150‰ to 200‰. Trehalase abundance was similar to G3LEA,showing a rapid decline under high salt stress 150‰ to 2000‰ (Fig 10).

### Localization of As-G1LEA and As-G3LEA

**Fig 11:**
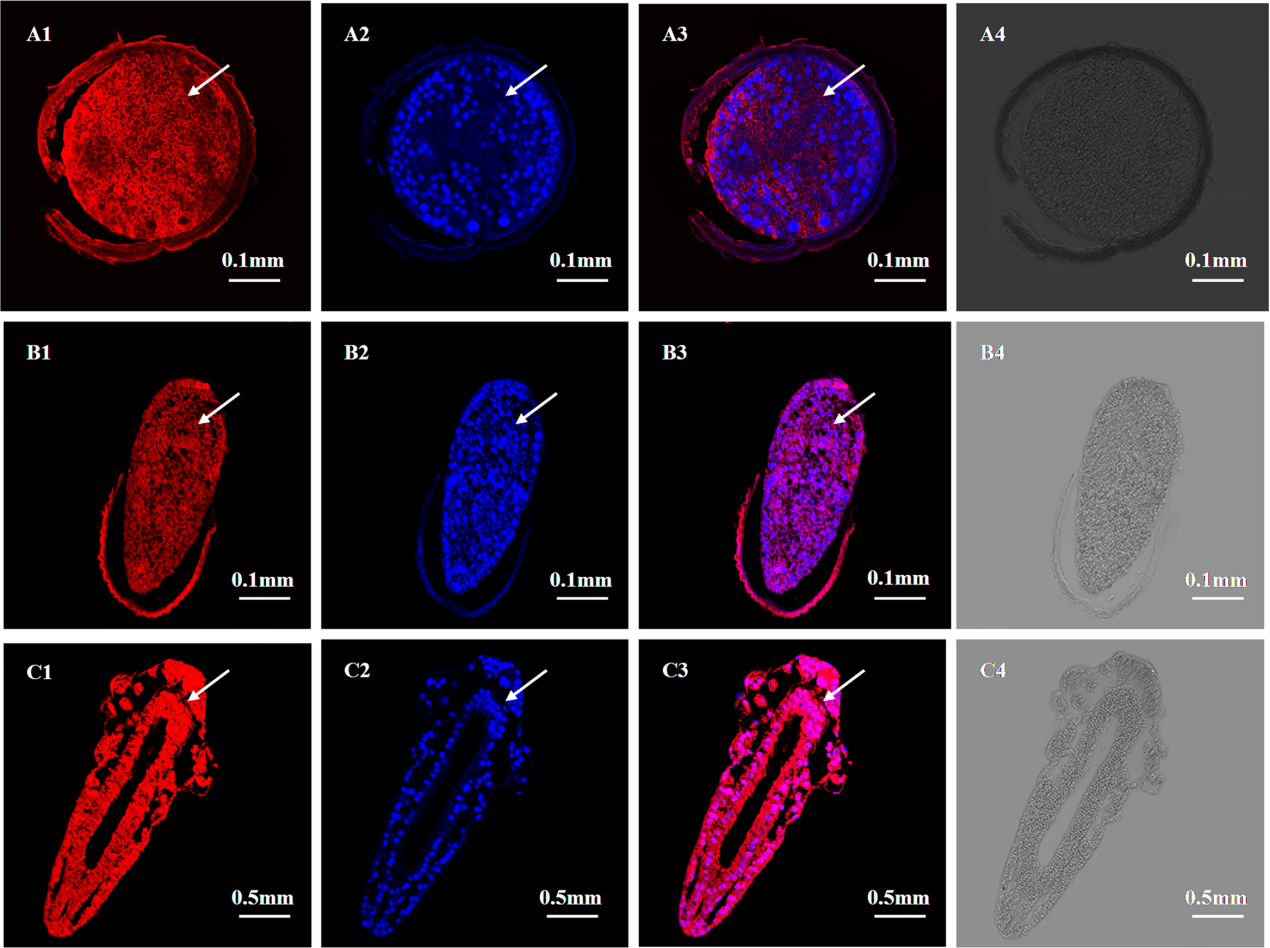
Immunolocalization of *As*-G1LEA at the Gastrula stage, embryonic stage and pre-adult stage in *Artemia sinica*. Paraffin sections of these stages were prepared for immunofluorescence microscopy as described in the experimental procedures. A1, B1 and C1 indicate single-labeling with polyclonal anti-G1LEA; A2, B2 and C2 indicate single-labeling with DAPI (cell nuclear blue fluorescent probe); A3, B3 and C3 represent the image overlay of the samples dual-labeled with polyclonal anti-G1LEA and DAPI. A4, B4 and C4 represent the image overlay of control group samples single-labeled with the secondary antibody. The arrows indicate parts with a positive signal.

**Fig 12:**
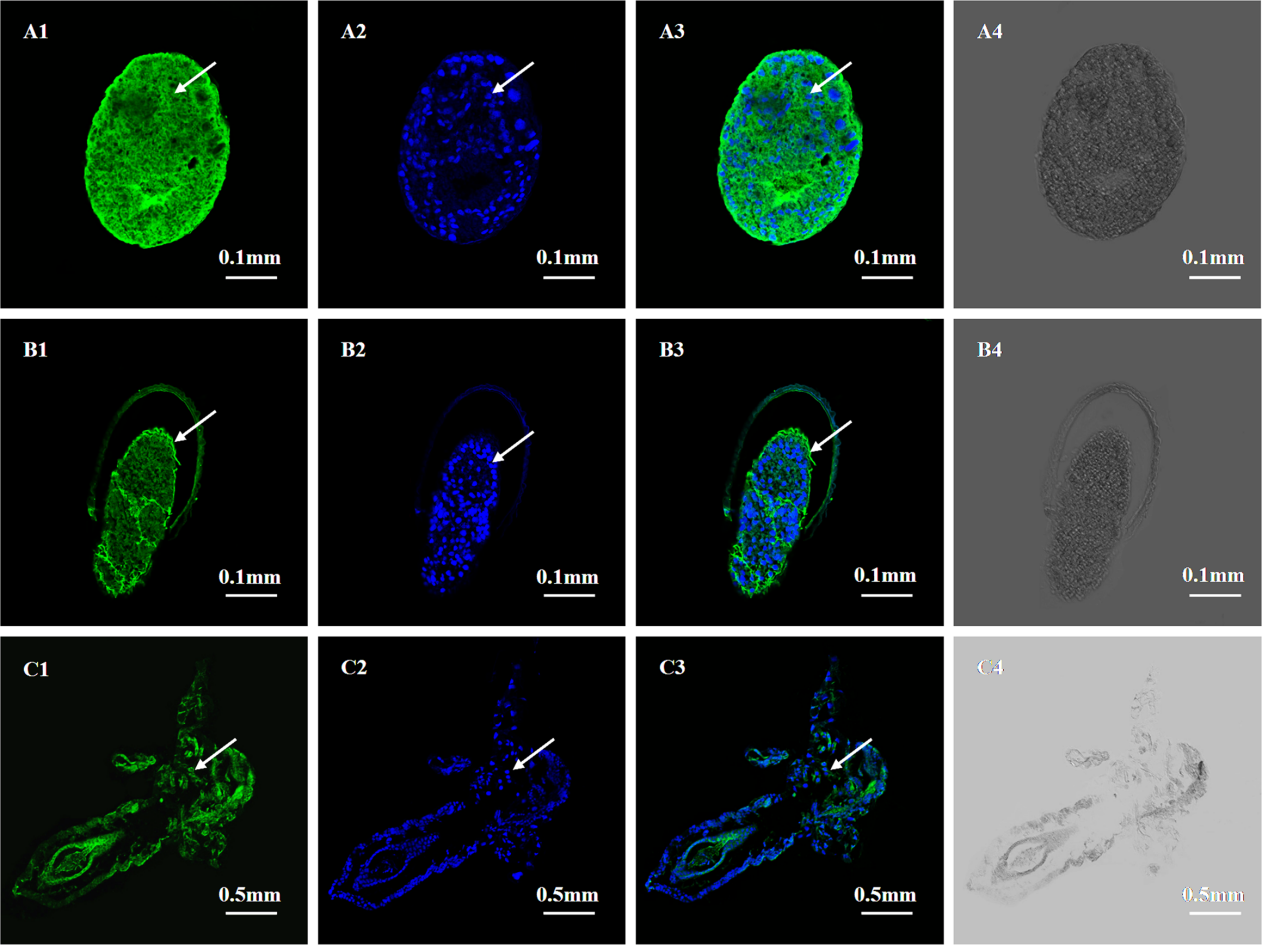
Immunolocalization of *As*-G3LEA at the Gastrula stage, embryonic stage and sub-adult stage in *Artemia sinica*. Paraffin sections of these stages were prepared for immunofluorescence microscopy, as described in the experimental procedures. A1, B1 and C1 indicate single-labeling with polyclonal anti-G3LEA; A2, B2 and C2 indicate single-labeled with DAPI (cell nuclear blue fluorescent probe); A3, B3 and C3 represent the image overlay of the samples dual-labeled with polyclonal anti-G3LEA and DAPI. A4, B4 and C4 represent the image overlay of control group samples single-labeled with the secondary antibody. The arrows indicate parts with a positive signal.

To analyze the expression localization of *As-*G1LEA and *As*-G3LEA proteins in different stages of embryo development of *A*. *sinica*, we detected the locations of *As*-G1LEA (red signal) and *As*-G3LEA (green signal) in embryos (0h, 15h) and pre-adults (3d) using immunofluorescence microscopy. The results showed that in the embryonic stage (0h), the *As*-G1LEA and *As*-G3LEA fluorescence signals were coincident with the position marked by DAPI probe and could be detected nearly throughout the whole embryo (Fig 11A, Fig 12A). At 15 h (umbrella stage), the fluorescence signals extended gradually from the head to the tail (Fig 11B, Fig 12B). In pre-adult stages (3d), *As*-G1LEA and *As*-G3LEA were detected in all parts of the specimen (Fig 11C, Fig 12C). No signal was detected in the control group without the anti- G1LEA antibody, anti-G3LEA antibody and DAPI probe.

## Discussion

In this study, we isolated the full-length cDNA sequences of *g1lea* and *g3lea* from *A*. *sinica* for the first time. The *As- g1lea* gene encodes a putative protein of 182 amino acids. It had no signal peptide or transmembrane domain, indicating that it is not a secreted or transmembrane protein. The hydrophobicity analysis of *As-g1lea* indicated that the protein is extremely hydrophilic. It is mainly composed of a repeating 20 amino acid motif, which is similar to that in plants. The motif is arranged in tandem with one to four copies in plants. However, in *Artemia franciscana*, it contains eight 20-mer repeats, which is the same in *A*. *sinica* [12]. The hydrophilic 20-mer motif determines the protein flexibility and structural stability [30]. This is a unique structure and might be the reason for the protein playing a special role during water shortage.

*As-g3lea* encodes a putative protein of 364 amino acids. It also had no signal peptide or transmembrane domain, indicating that it is not a secreted or transmembrane protein. The hydrophobicity analysis of *As-G3LEA* indicated that the protein is hydrophilic. As a consequence of the changes introduced in the repeating11-mer amino acid motif, group 3 members are quite diverse [17]. The secondary structure might depend on the drying rate and these structural transitions are fully reversible [19]. The different approaches used to elucidate the function of group 3 proteins suggest that they contribute to counteracting the damage produced by water loss [11].

According to the qPCR and western blotting results, the high levels of *As*-G1LEA at 0h might be derived from the accumulation of cell in that phase that are stored at low temperatures before the beginning of embryonic development. Then, the protein expression level dropped significantly from 0h to 15h in the embryo; in the adult it continued to show an extremely low expression level. This indicated that *As*-G1LEA might have no effect on the timing of embryo development in *Artemia* [6]. *As*-G3LEA is somewhat similar to *As*-G1LEA; the expression level at the embryonic period was higher than the adult. But unlike G1LEA at 3d, there was little increase. 3d is the important phase when the larvae develop into adults [31]. Therefore, we cannot rule out a potentially important role of *As*-G3LEA proteins in early embryo development. We used the expression of trehalase, which is capable of degrading trehalose, as a measure of the intracellular trehalose level. The increased expression of trehalase over time meant that the intracellular trehalose content decreased. Trehalase expression gradually increased from 0h to 20h, indicating that the trehalose that accumulated in diapause stage is substantially decomposed by trehalase until its expression decreased after 40h. Trehalase can degrade trehalose to the glucose involved in energy metabolism. Trehalose may provide energy for early embryonic development. Judging from the experimental results, *As*-G1LEA and *As*-G3LEA proteins have little effect on the early embryonic development of *A*. *Sinica*.

The experimental qPCR and western blot results showed that the levels of As-G1LEA and As-G3LEA proteins show a similar trend of gradual increase as the temperature decreases. By contrast, as the temperature falls, the abundance of trehalase decreases. Trehalose can protect proteins and cellular membranes, avoiding the damage that results in a variety of stress conditions, such as desiccation, dehydration, cold and oxidation [32]. LEA proteins most likely stabilize outer mitochondrial membrane and when trehalose was present on both sides of the lipid bilayer the stabilization was much greater [24]. Similarly, LEA proteins have the same trend with trehalose, which suggested that both G1LEA and G3LEA proteins might participate in cold tolerance. The G1LEA proteins protected citrate synthase against desiccation and accompanied by significant increases in trehalose in vitro [16]. AfrLEA3 is thought to protect mitochondria against drying damage, with trehalose having a supplementary role [10,33]. Recent studies suggested that trehalose and LEA proteins work synergistically during desiccation [34,19]. Sugar glasses form a structural network to increase cellular resistance to water loss, which might be related with LEA proteins, and trehalose is well known component of biological glasses [35,36,37]. In addition, LEAs work synergistically with trehalose to form sugar glasses to help cells against the cold. In this process, LEA proteins may act as “molecular shields” to prevent aggregation of denatured proteins caused by a cold-induced desiccation. This might also lead to the expression levels of two LEA proteins being much higher than the control group at 5C [38]. These two LEA proteins might participate in resistance against drying caused by low temperature, with trehalose’s help.

Salinity of 50‰ is the optimum for survival of the adult *Artemia*; however, *As*-G1LEA level dropped dramatically drop from 50‰ and was maintained a low a level until 200‰, which suggested that *As*-G1LEA proteins might not participate in osmotic regulation. There were some differences between *As*-*g1lea* and *As*-*g3lea* expressions. *As*-*g3lea* transcript levels began to decrease reaching their lowest at 200‰ might reflect and effect of high salt on the life activities of brine shrimp. However, in the experimental group, trehalase expression gradually decreased compared with the control group. This showed that as the salinity increases, trehalose was still accumulating. One of the possible reasons is that too high salinity hinders protein synthesis, or more likely, *As*-G3LEA proteins may not work with trehalose in osmotic regulation.

The immunofluorescence microscopy results showed that the *As*-G1LEA protein and *As-*G3LEA protein were located in different tissues and organs of *A*. *sinica*. The two LEA proteins were expressed in the whole body, and from embryo to the adult, the expression of LEA proteins is sustained. The result showed that the regulation of LEA proteins may not be limited by the developmental stage or organ specificity. *A*. *sinica* against to low temperature is most likely to be systemic. LEA proteins are widely distributed in various parts of plant organs and cells, while most LEA protein families have a unique subcellular localization; members of the *As*-G3LEA proteins are widely distributed in the cytosol, mitochondria, plastid, ER and pexophagosome [39]. The subcellular distribution of LEA proteins highlights the requirement for each cellular part to cope with desiccation or cold stress, coincidence with the conclusion of Adrien et al. in 2014.

## Conclusion

Animal against adverse environmental mechanism is very complex. In this study, the *As-g1lea and As-g3lea* transcript was cloned for the first time from *A*. *sinica* by using RACE technology. Expression location analysis showed the expression of *As*-G1LEA and *As*-G3LEA proteins in different positions and various developmental periods of *A*. *sinica*, which proved there is no organ-specificity for these proteins. Trehalose is stress tolerance related proteins, and real-time qPCR and Western blot results show the expression levels of these two LEA proteins in *A*. *sinica* during early embryonic development stages were significantly higher than in the adult, illustrating that *As*-G1LEA and *As*-G3LEA proteins may have little effect on the early embryonic development of *A*. *sinica*. The expression level of LEA proteins expressions matched the trend of accumulation of trehalose, as judged by trehalase’s reaction to the temperature challenge show that the potential roles of LEA proteins under temperature stress and high salinity in *A*. *sinica*. LEA proteins may help cell fight against with multiple damage due to water loss. This study provides indirect evidence for the potential functions of LEA proteins in animals and a molecular mechanism of the stress tolerance exerted by LEA proteins in *A*. *sinica*.

## Supporting Information

S1 Fig Raw WB photograph of Fig 6-G1LEA. (JPG)

S2 Fig Raw WB photograph of Fig 7-G3LEA. (JPG)

S3 Fig Raw WB photograph of Fig 8-G1LEA. (JPG)

S4 Fig Raw WB photograph of Fig 8-G3LEA. (JPG)

S5 Fig Raw WB photograph of Fig 8-GAPDH. (JPG)

S6 Fig Raw WB photograph of Fig 8-Trehalase. (JPG)

S7 Fig Raw WB photograph of Fig 9-G1LEA. (JPG)

S8 Fig Raw WB photograph of Fig 9-G3LEA. (JPG)

S9 Fig Raw WB photograph of Fig 9-GAPDH. (JPG)

S10 Fig Raw WB photograph of Fig 9-Trehalase. (JPG)

S11 Fig Raw WB photograph of Fig 10-G1LEA. (JPG)

S12 Fig Raw WB photograph of Fig 10-G3LEA. (JPG)

S13 Fig Raw WB photograph of Fig 10-GAPDH. (JPG)

S14 Fig Raw WB photograph of Fig 10-Trehalase. (JPG)
