# Supplementary figures and images for: The Potential Roles of the G1LEA and G3LEA Proteins in Early Embryo Development and in Response to Low Temperature and High Salinity in Artemia sinica
Source: PLoS One. 2016 Sep 7;11(9):e0162272. doi: 10.1371/journal.pone.0162272 (PMC5014412; doi:10.1371/journal.pone.0162272)

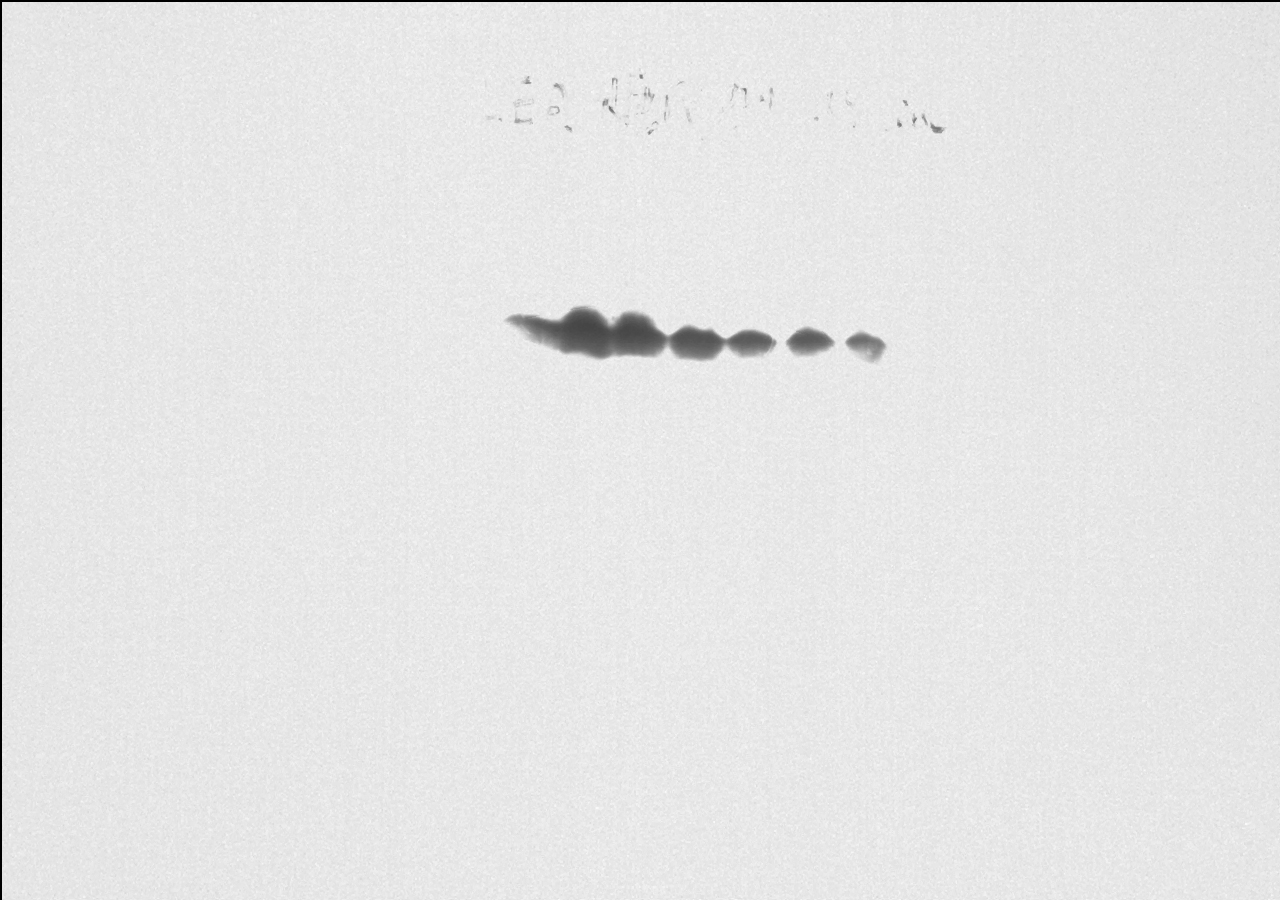

Supplement: S1 Fig — (JPG) [file pone.0162272.s001.jpg]

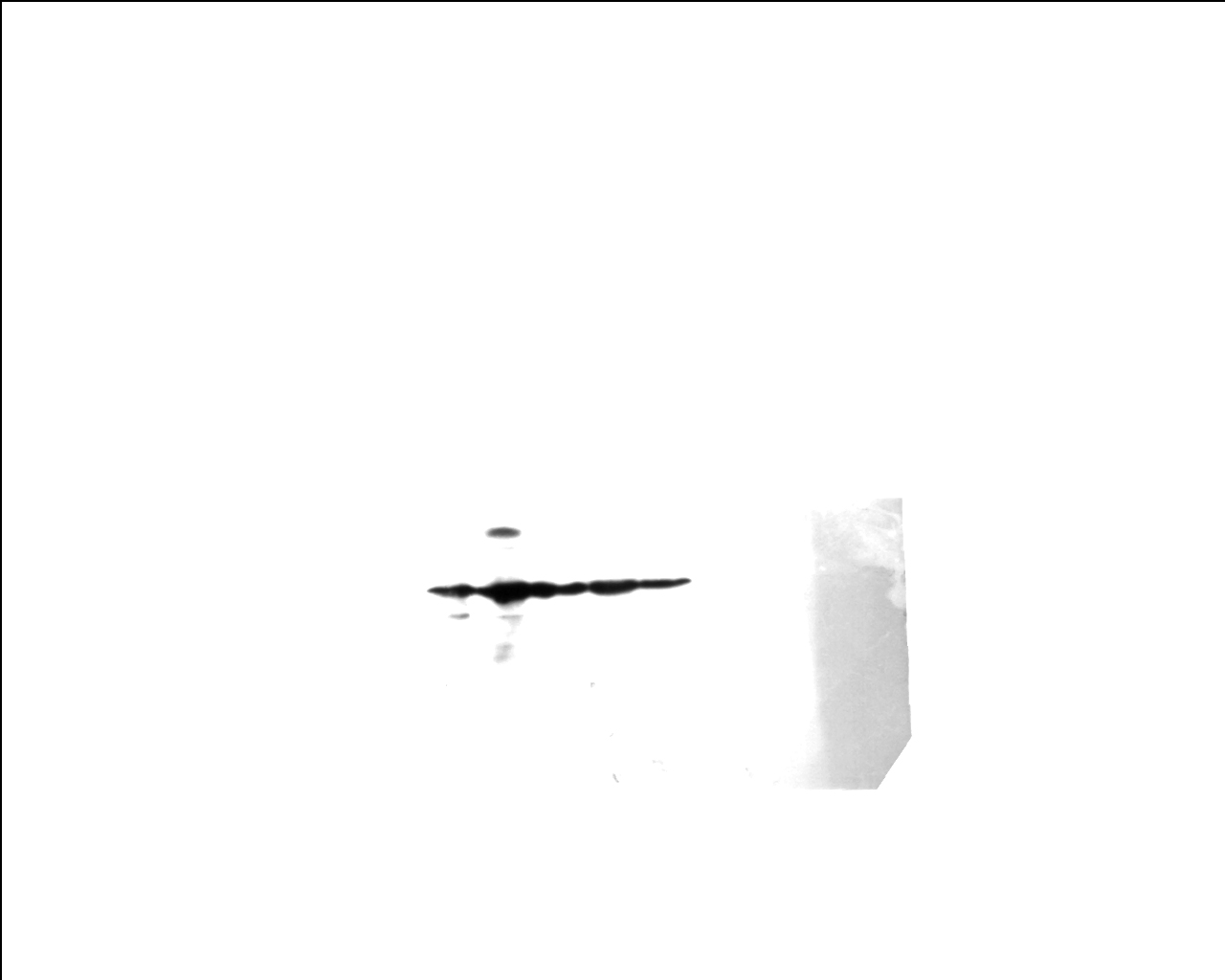

Supplement: S2 Fig — (JPG) [file pone.0162272.s002.jpg]

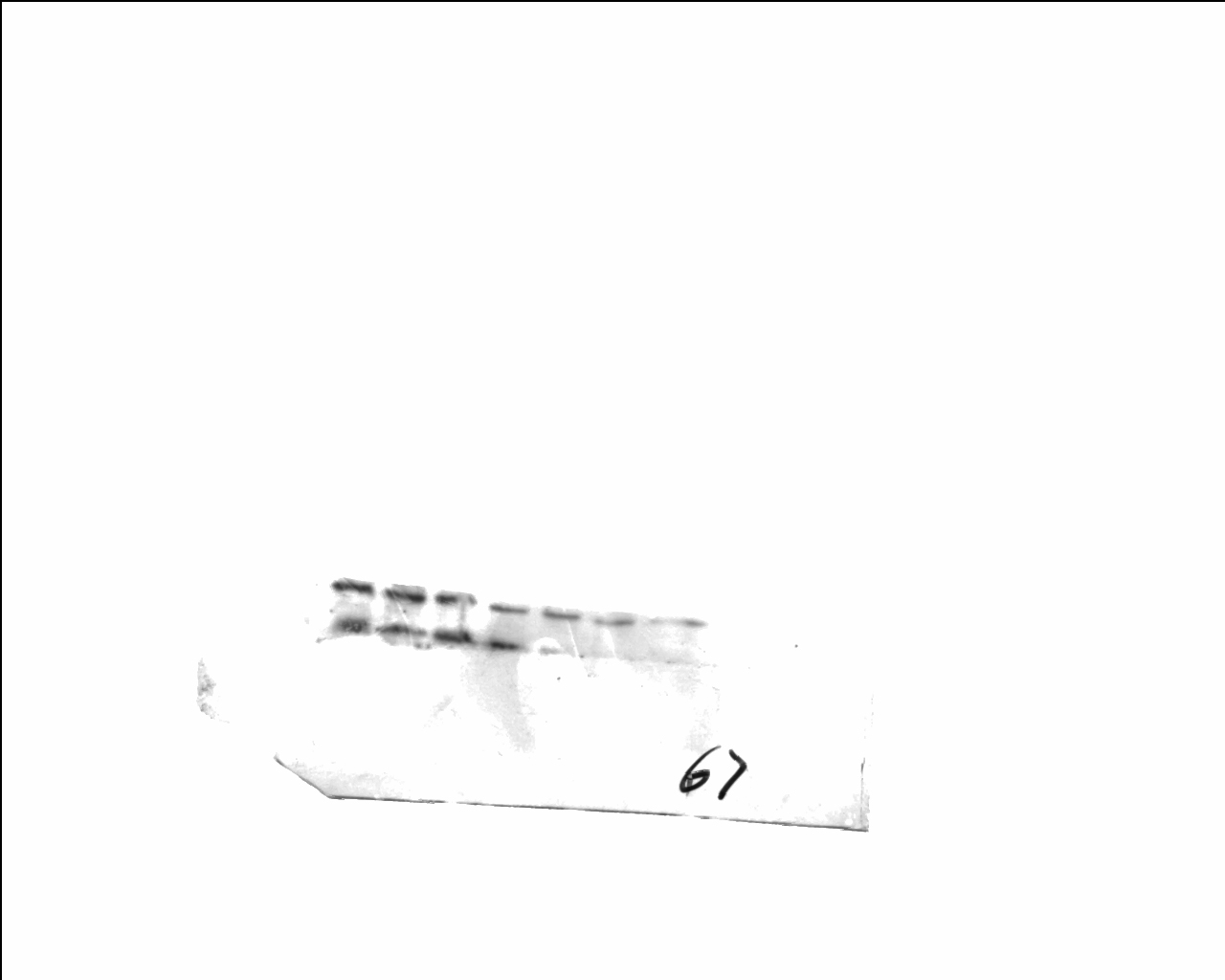

Supplement: S3 Fig — (JPG) [file pone.0162272.s003.jpg]

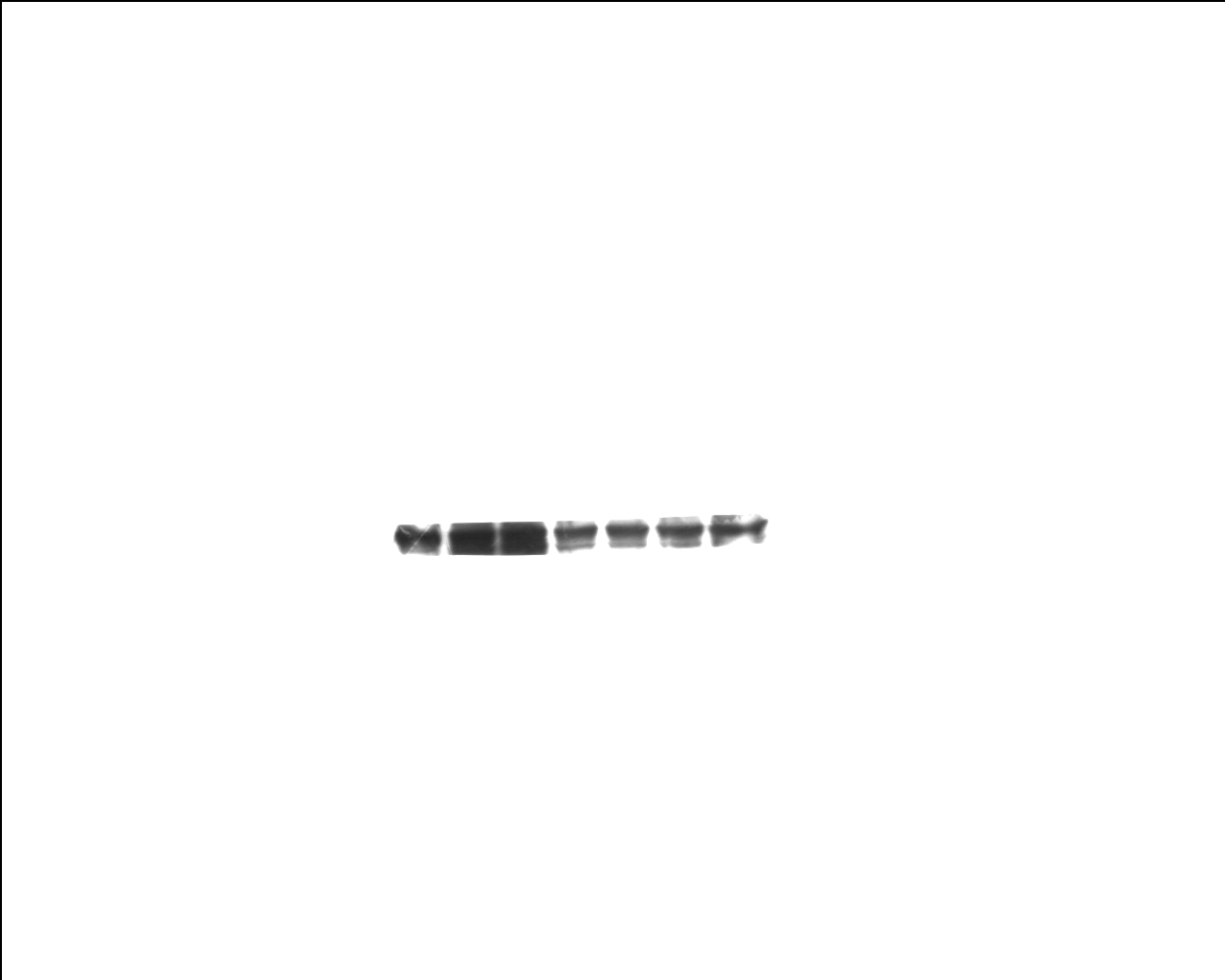

Supplement: S4 Fig — (JPG) [file pone.0162272.s004.jpg]

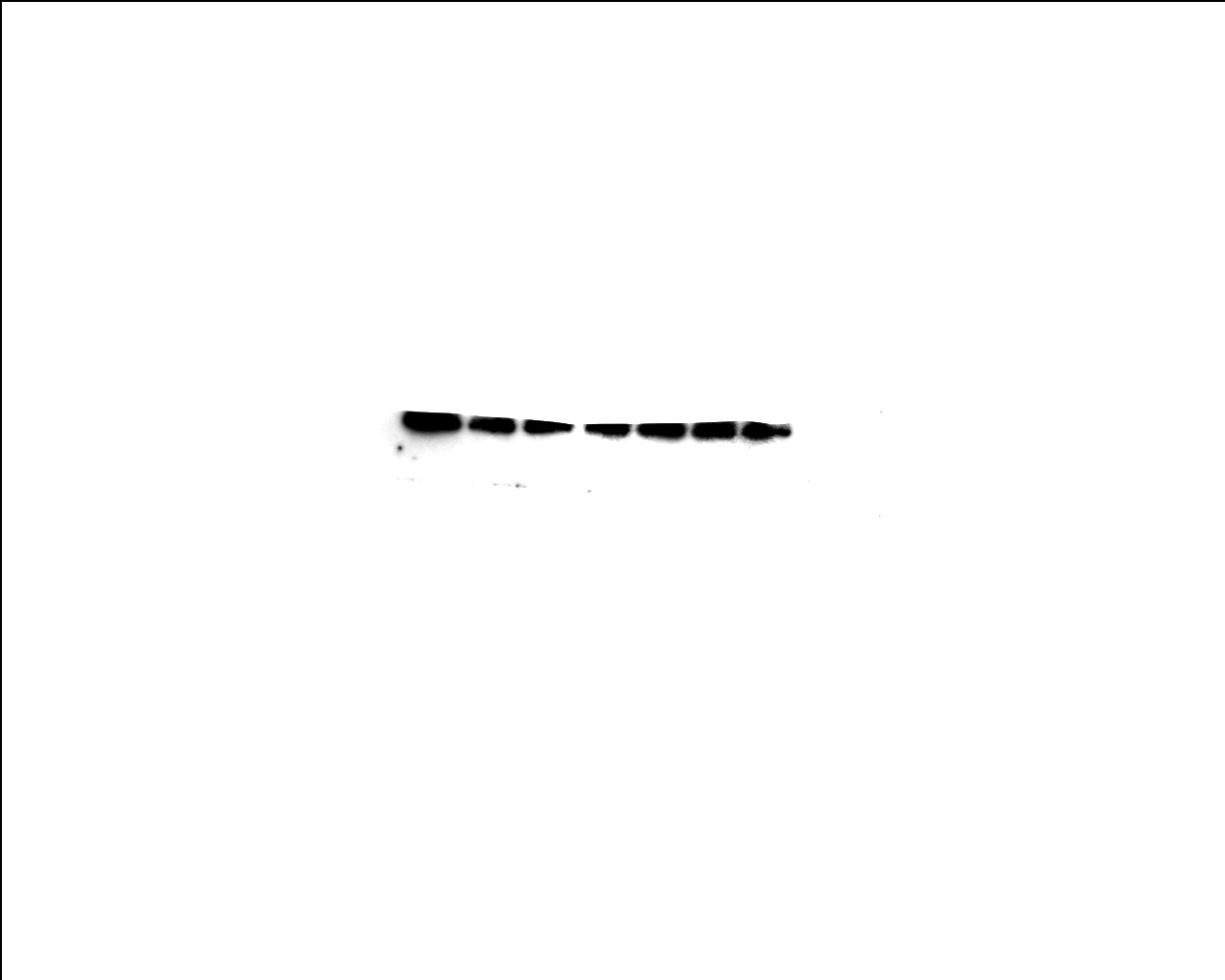

Supplement: S5 Fig — (JPG) [file pone.0162272.s005.jpg]

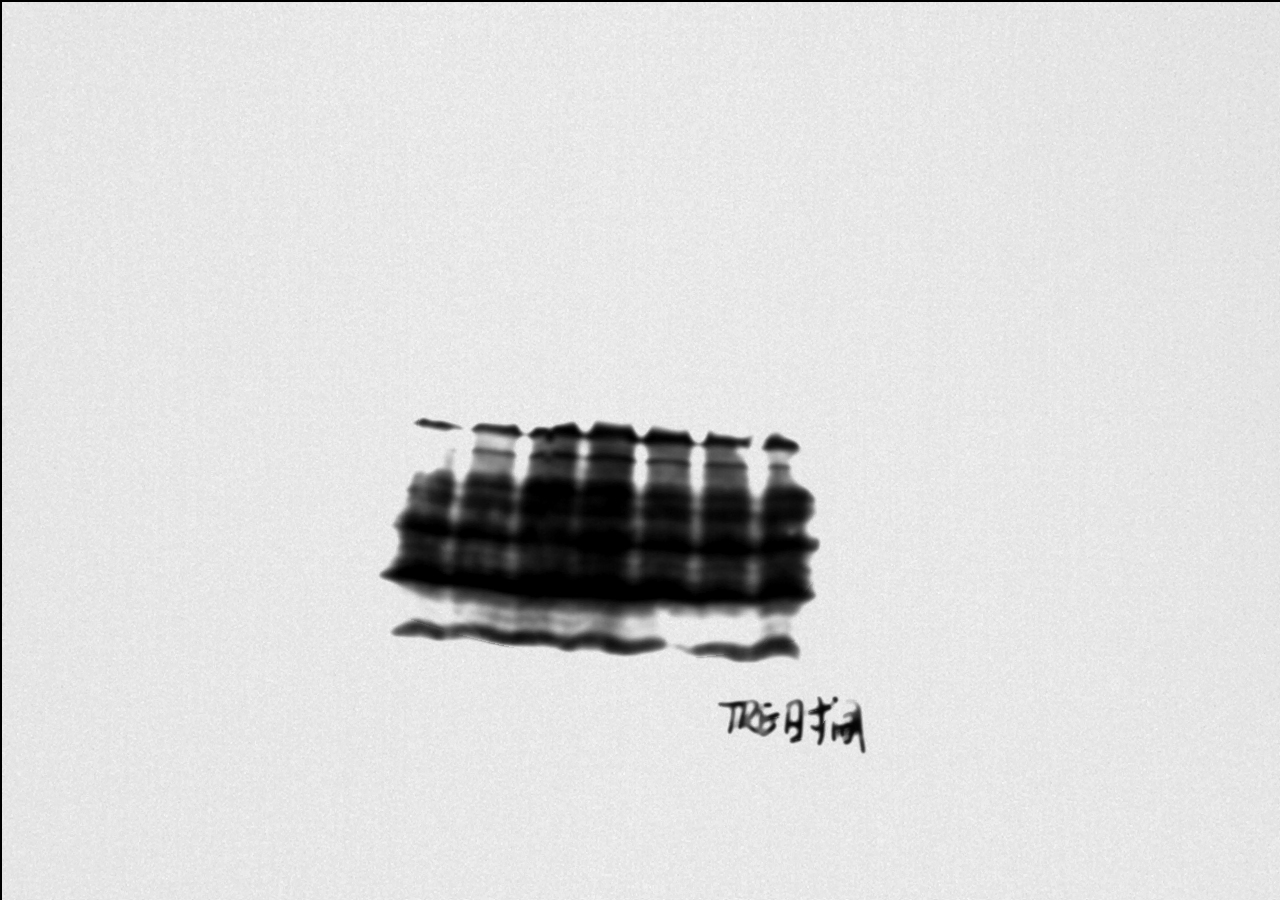

Supplement: S6 Fig — (JPG) [file pone.0162272.s006.jpg]

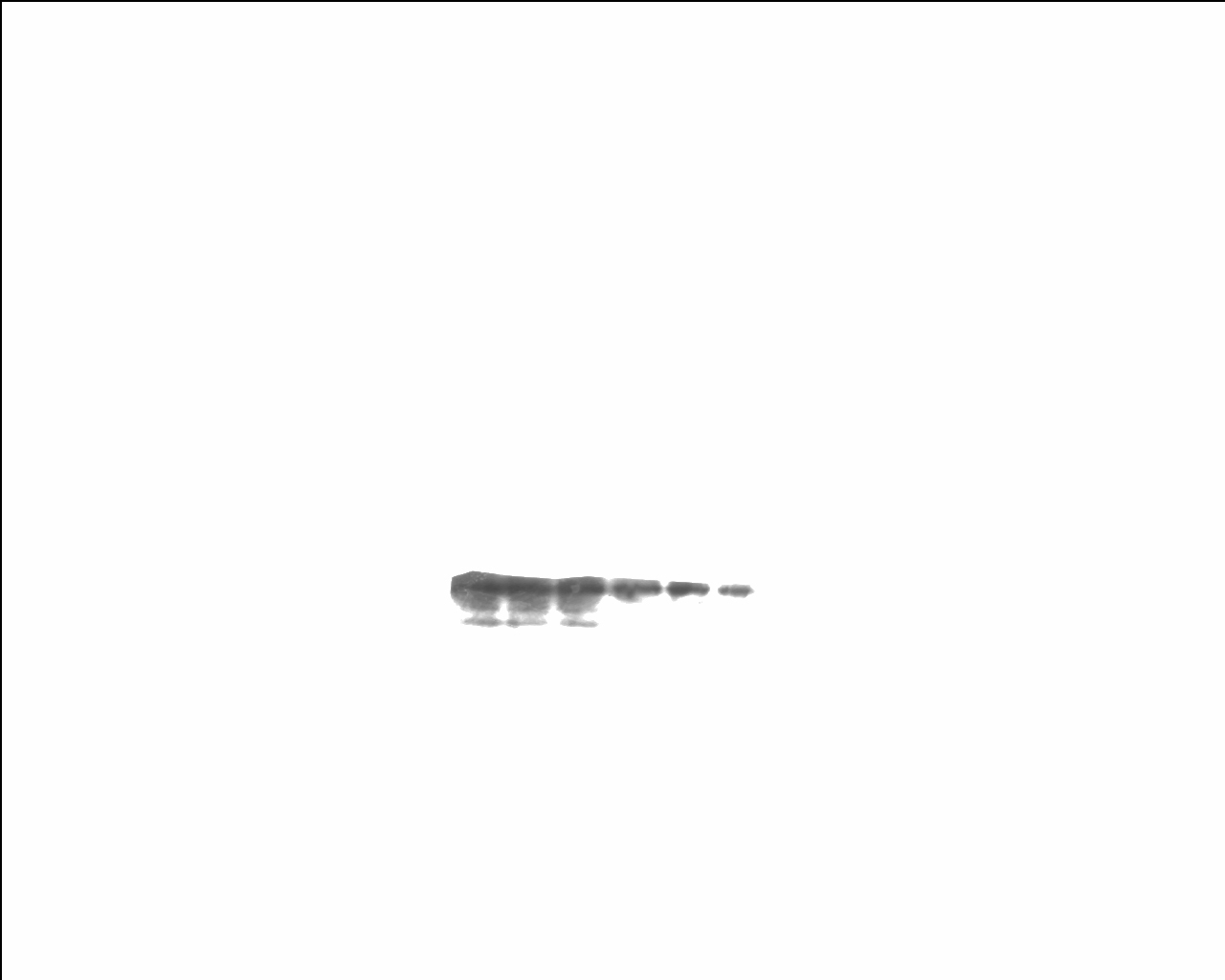

Supplement: S7 Fig — (JPG) [file pone.0162272.s007.jpg]

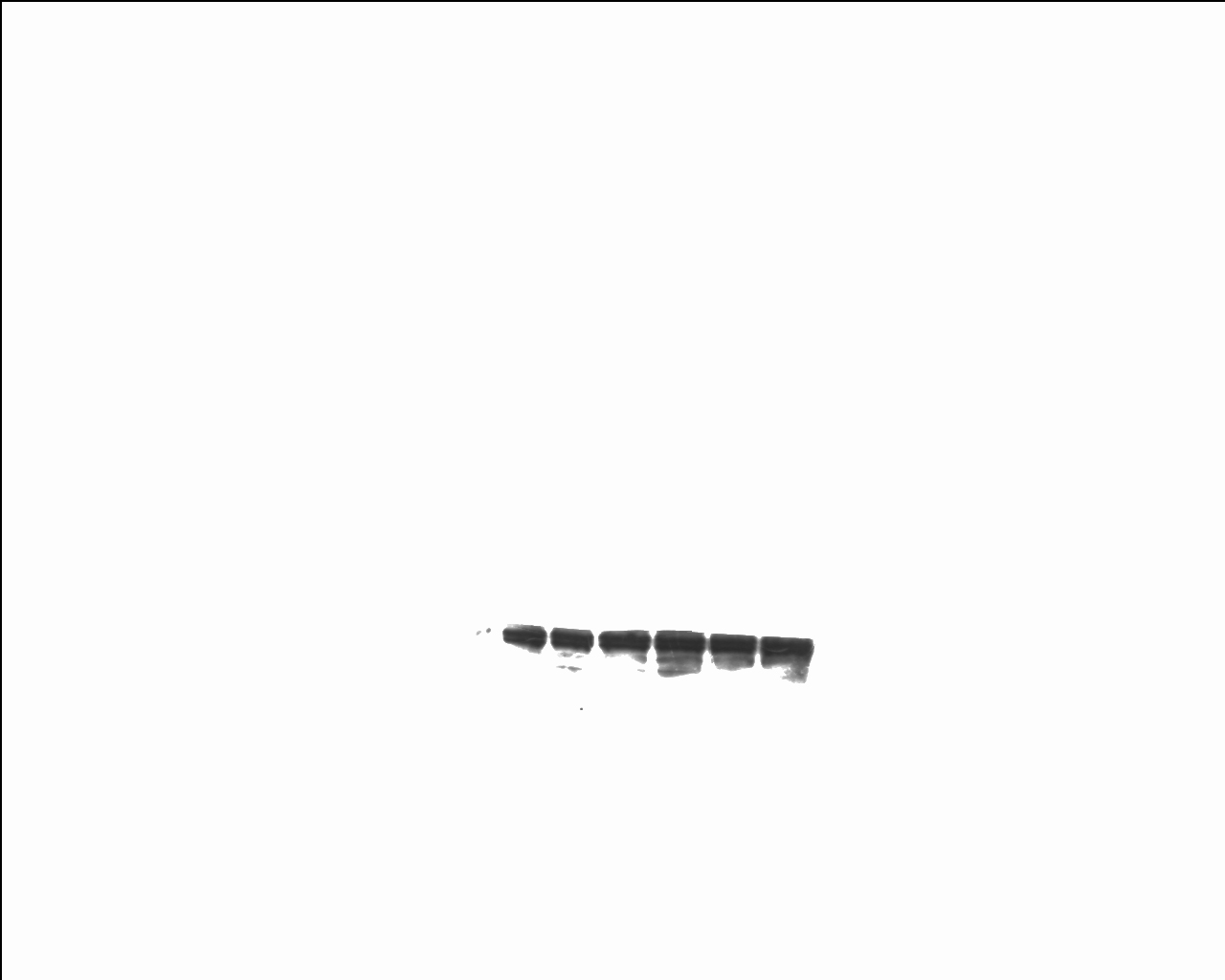

Supplement: S8 Fig — (JPG) [file pone.0162272.s008.jpg]

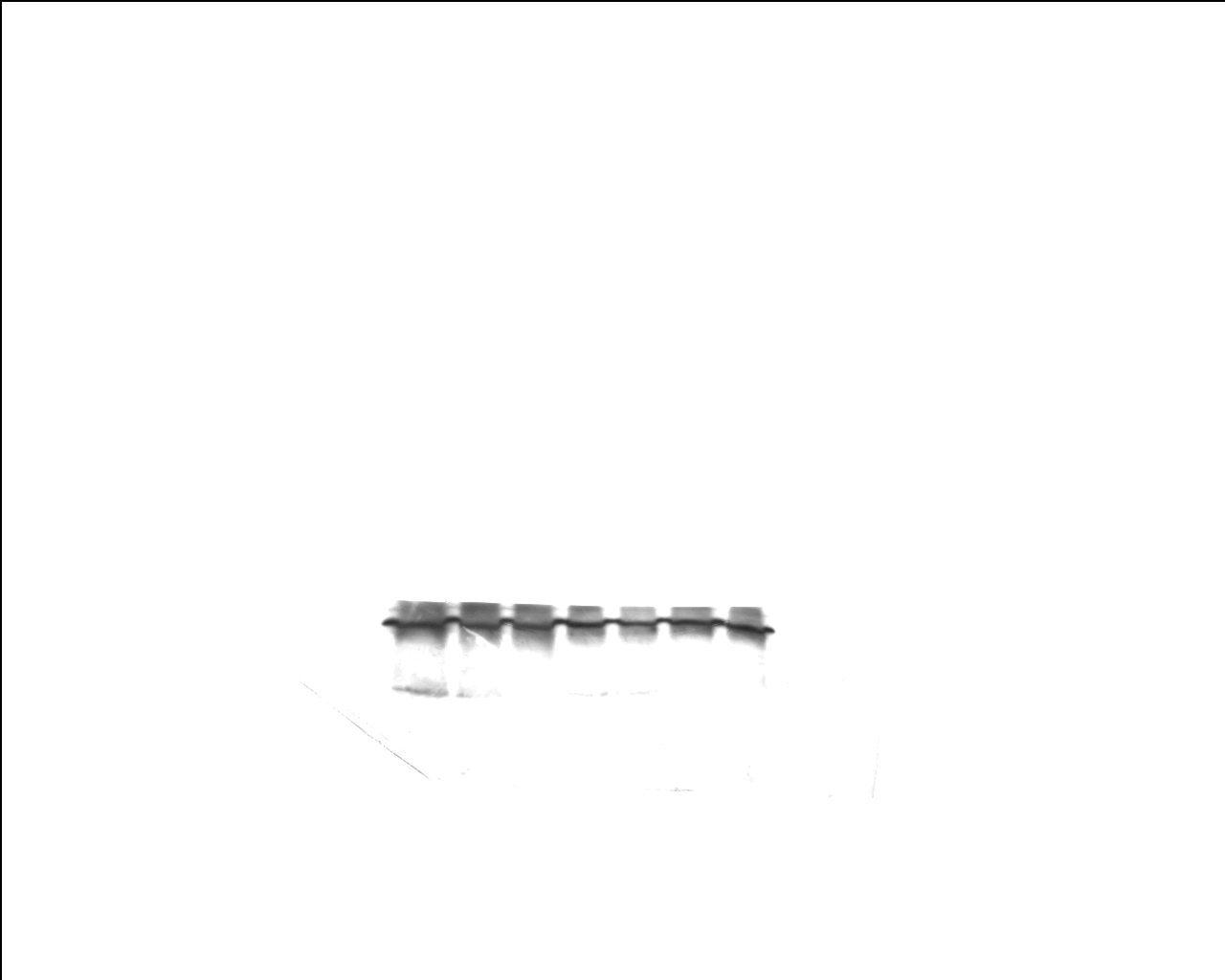

Supplement: S9 Fig — (JPG) [file pone.0162272.s009.jpg]

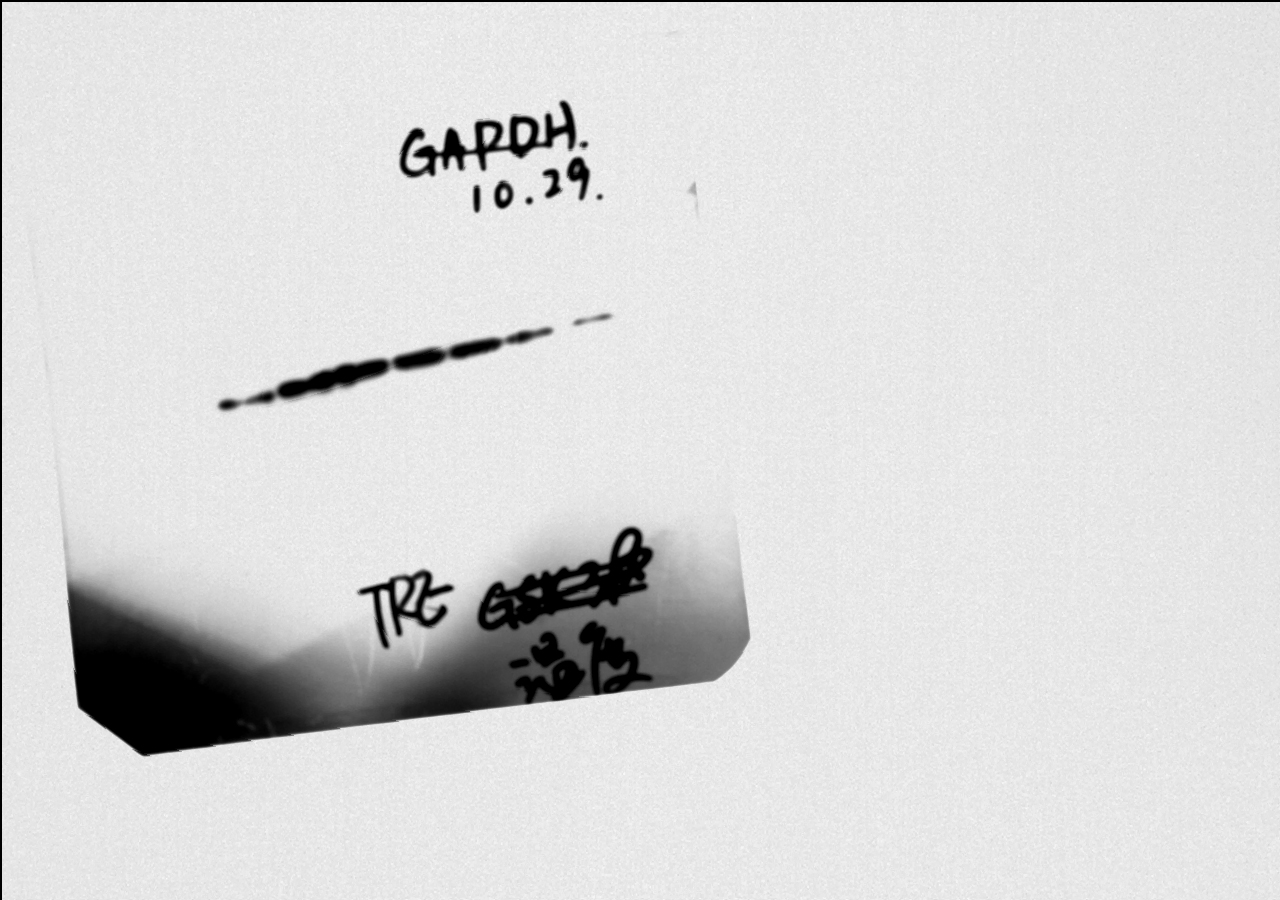

Supplement: S10 Fig — (JPG) [file pone.0162272.s010.jpg]

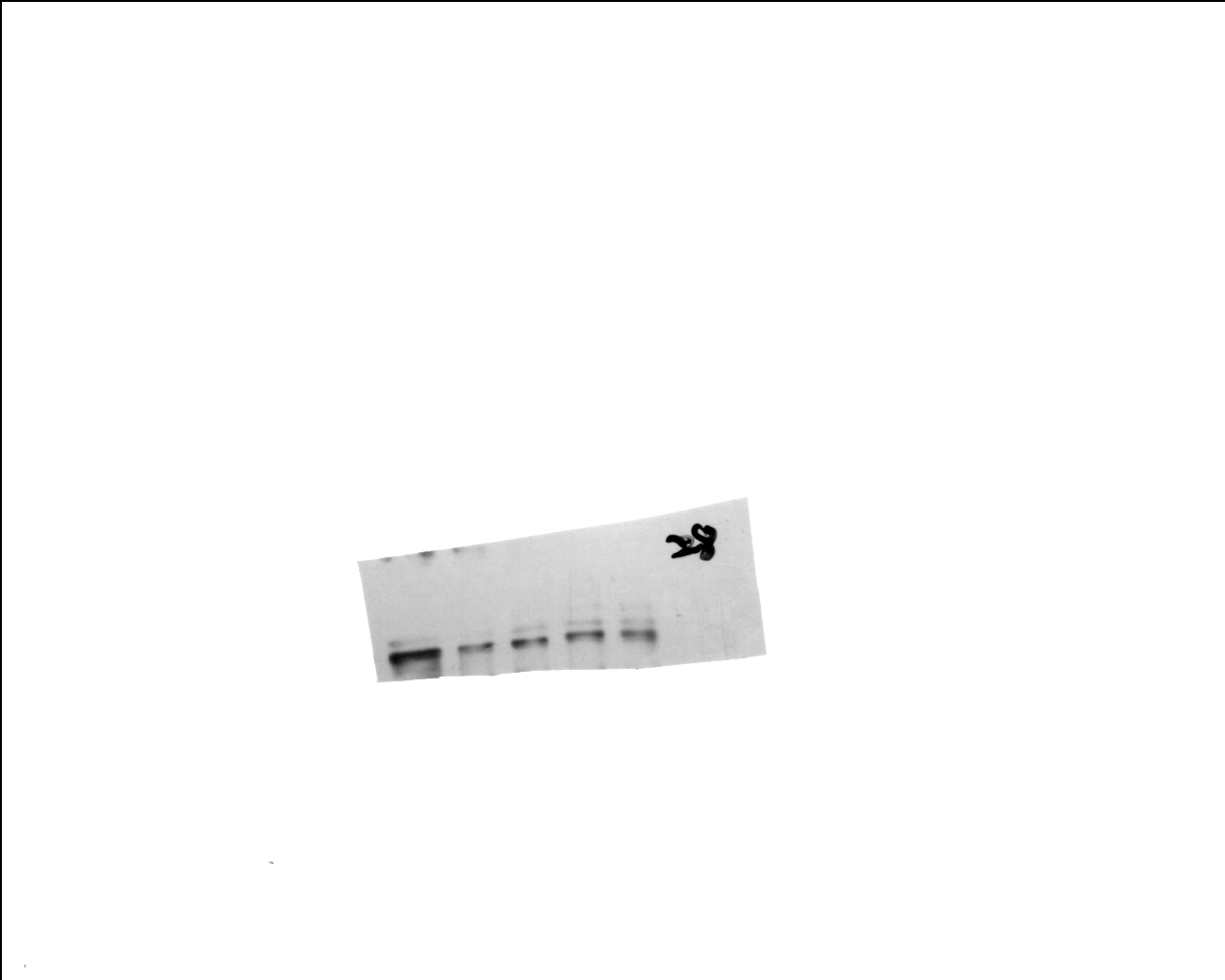

Supplement: S11 Fig — (JPG) [file pone.0162272.s011.jpg]

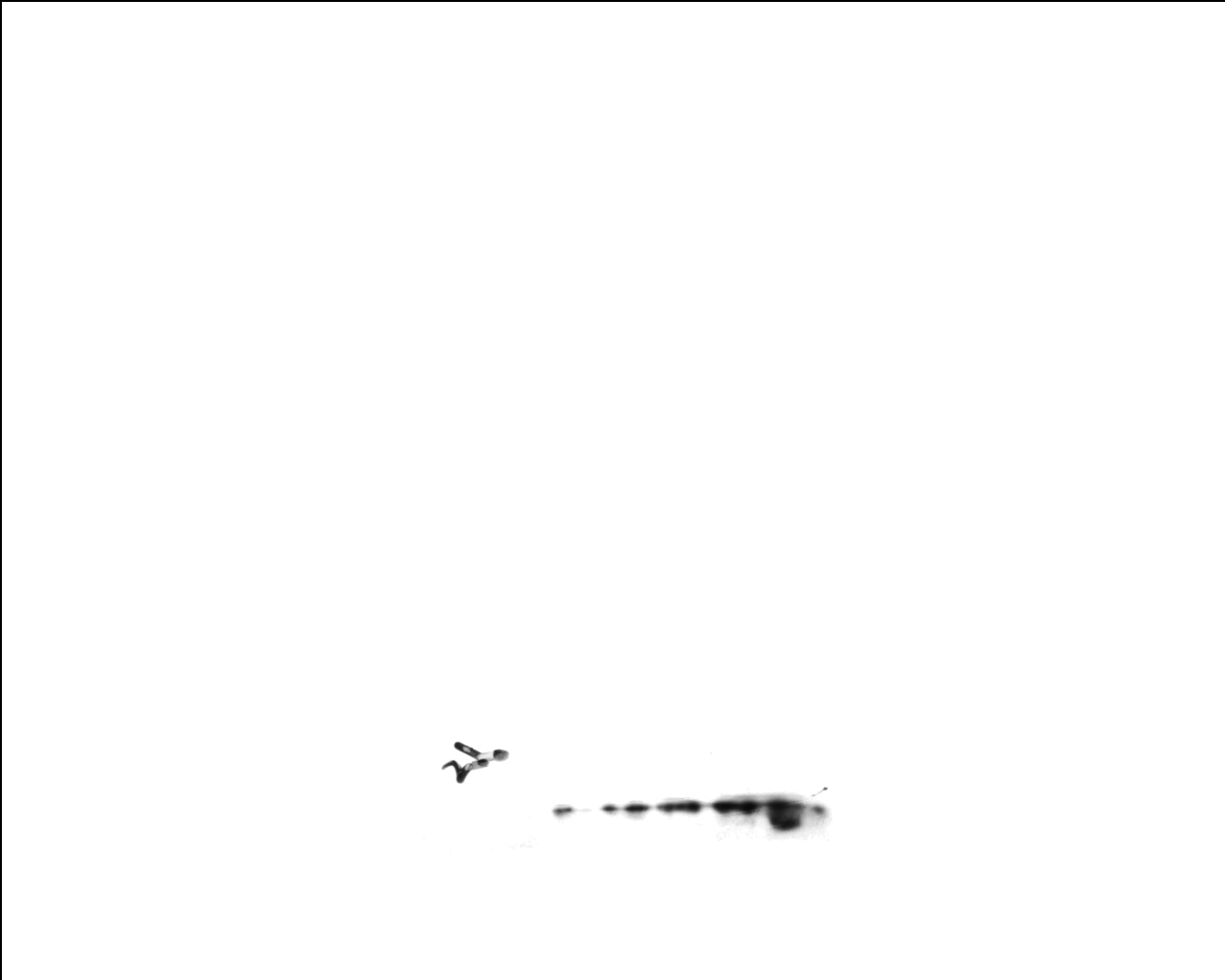

Supplement: S12 Fig — (JPG) [file pone.0162272.s012.jpg]

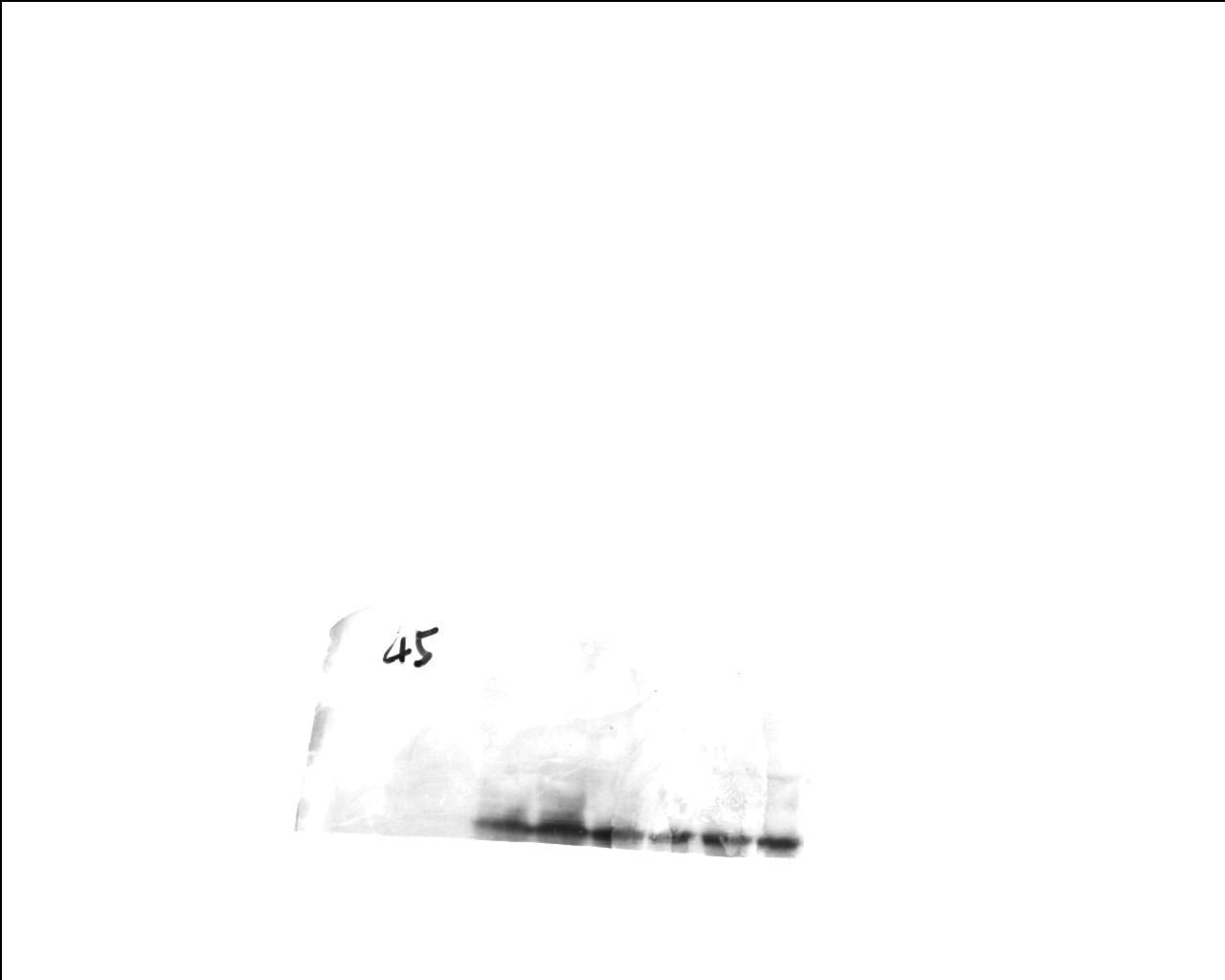

Supplement: S13 Fig — (JPG) [file pone.0162272.s013.jpg]

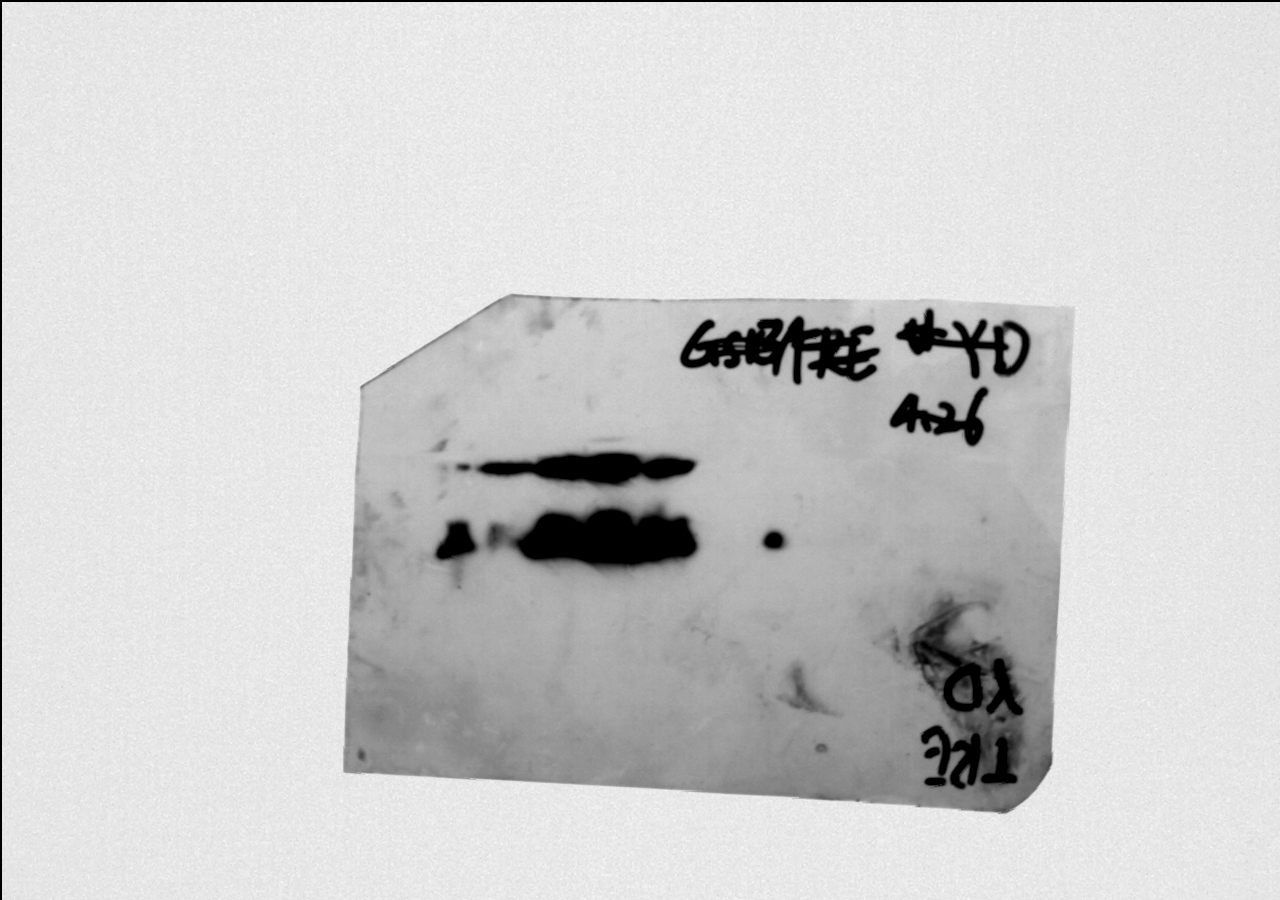

Supplement: S14 Fig — (JPG) [file pone.0162272.s014.jpg]
